# Label-Free Digital Holographic Microscopy for In Vitro Cytotoxic Effect Quantification of Organic Nanoparticles

**DOI:** 10.3390/cells11040644

**Published:** 2022-02-12

**Authors:** Kai Moritz Eder, Anne Marzi, Álvaro Barroso, Steffi Ketelhut, Björn Kemper, Jürgen Schnekenburger

**Affiliations:** Biomedical Technology Center, University of Muenster, Mendelstraße 17, D-48149 Muenster, Germany; kai.eder@uni-muenster.de (K.M.E.); anne.marzi@uni-muenster.de (A.M.); alvaro.barroso@uni-muenster.de (Á.B.); ketelhut@uni-muenster.de (S.K.)

**Keywords:** digital holographic microscopy quantitative phase imaging, nanoparticles, cytotoxicity, in vitro, cell culture, dry mass, WST-8 cell viability assay

## Abstract

Cytotoxicity quantification of nanoparticles is commonly performed by biochemical assays to evaluate their biocompatibility and safety. We explored quantitative phase imaging (QPI) with digital holographic microscopy (DHM) as a time-resolved in vitro assay to quantify effects caused by three different types of organic nanoparticles in development for medical use. Label-free proliferation quantification of native cell populations facilitates cytotoxicity testing in biomedical nanotechnology. Therefore, DHM quantitative phase images from measurements on nanomaterial and control agent incubated cells were acquired over 24 h, from which the temporal course of the cellular dry mass was calculated within the observed field of view. The impact of LipImage™ 815 lipidots^®^ nanoparticles, as well as empty and cabazitaxel-loaded poly(alkyl cyanoacrylate) nanoparticles on the dry mass development of four different cell lines (RAW 264.7, NIH-3T3, NRK-52E, and RLE-6TN), was observed vs. digitonin as cytotoxicity control and cells in culture medium. The acquired QPI data were compared to a colorimetric cell viability assay (WST-8) to explore the use of the DHM assay with standard biochemical analysis methods downstream. Our results show that QPI with DHM is highly suitable to identify harmful or low-toxic nanomaterials. The presented DHM assay can be implemented with commercial microscopes. The capability for imaging of native cells and the compatibility with common 96-well plates allows high-throughput systems and future embedding into existing experimental routines for in vitro cytotoxicity assessment.

## 1. Introduction

Nanotechnology is an emerging research field in medicine and pharmaceutical sciences. The physico-chemical advantages of nanoparticles are utilized for diagnosis of diseases, enhanced drug delivery, as well as for support in regenerative medicine [1,2]. During the last decade, the US Food and Drug Administration has approved the commercialization of more than 100 nanomedicine applications and products [3]. For example, inorganic nanoparticles like iron oxide and gold are applied as contrast agents [4,5] while, organic nanomaterials like polymeric or liposomal, as investigated in this study, can fulfill various pharmaceutical and medical functions, such as drug carriers, contrast agents, immunotherapies or gene editors [6,7]. Recently, the prominent role of medical nanotechnology in current science and medicine was highlighted by the lipid nanoencapsulation of the mRNA-based COVID-19 vaccine [8,9]. However, the advantageous properties of organic nanomaterials are also accompanied by complexities, which require extensive preclinical testing. In contrast to industrial nanomaterials, for which primarily risks to humans upon accidental exposure must be considered, for medical applications, the understanding of the entire impact of nanoparticles on cellular processes is essential for both effectiveness and safety [10,11].

Preclinical research and safety control of nanomaterials are commonly performed utilizing dye-based in vitro cell culture assays. For the development of new drugs, drug carriers, or contrast agents, the assessment of cytotoxicity and effects on cell viability and proliferation represents an essential starting point, which requires assay chemicals for quantitative endpoint determination. Well-known challenges of in vitro cell culture assays are interactions of optically active nanomaterials with colorimetric cytotoxicity assays, which require a careful selection of suitable test systems for accurate hazard and safety characterization [12,13]. Often, optical particle-assay interactions limit the applicable concentrations of the tested nanomaterial and the determination of low effect levels of low-toxicity nanomaterials [14,15]. In addition, fluorescence labeling-based microscopy is widely used for mechanistic analysis of cellular responses to small molecules and nanomaterials. Although fluorescence techniques show high specificity, the involved laboratory resources can be high, and, similar to colorimetric methods, nanomaterial-assay interferences have to be carefully considered [12,16]. In particular, due to the various possible nanomaterial-assays interactions identified in several studies, results of colorimetry and label-based in vitro cytotoxicity studies on nanomaterials were found inconsistently and sometimes even contradictory [17,18]. These findings create the demand for new methods for the analysis of cellular responses to organic nanoparticles, which are insensitive to interactions with the tested nanomaterial and can be combined with other biochemical analysis methods downstream [19,20].

In this context, quantitative phase imaging (QPI) provides time-resolved in vitro assays for nanoparticle effect quantifications by label- and dye-free extraction of the cellular dry mass from quantitative phase images of native cell populations [21]. Recently, QPI has received increased attention due to various scientific and technological advances, in particular by improvements in optical hardware and computational power [21]. Various studies have demonstrated QPI to be a versatile tool for minimally invasive label-free imaging of biological specimens and time-resolved cellular analysis in preclinical biomedical setups [22]. This includes wound healing assays [23], cytometry [24,25], tumor drug resistance analysis [26], histopathology [27], and red blood cell phenotyping, e.g., in anemias [28] as well as cytotoxicity testing [29,30,31]. Engineered nanoparticles not intended for medical use have also been tested in a QPI-based cytotoxicity assay [32]. In contrast to many established cell viability and proliferation assays that measure specific endpoints after strict incubation times with the test material, the minimally invasive character of QPI allows for long-term time-resolved simultaneous assessment of cellular proliferation, cell death, and other morphology associated cellular processes. In addition to biophysical parameters like dry mass development [33,34], single-cell morphology and migration could be extracted from QPI images. These earlier results indicate QPI as potent and multimodal assay readout method.

In this study, digital holographic microscopy (DHM [35]), an interferometric variant of QPI, is explored as in vitro assay to quantify the effects of organic nanoparticles in development for medical use on four eukaryotic cell lines. To consider features and cellular diversity of different organs of origin, we incubated RAW 264.7 macrophages, NIH-3T3 fibroblasts, NRK-52E kidney cells, and RLE-6TN lung epithelial cells, representing different origin organs, with different concentrations of nanomaterials, digitonin as cytotoxicity control, as well as cell culture medium control and subsequently imaged the cells in a DHM system equipped with a stage-top incubator over 24 h. The resulting QPI image series were evaluated for changes in dry mass development as a readout for the proliferation of the investigated cells. In parallel, a widely used WST-8 cell viability assay was performed with the same cell lines and nanomaterials to compare metabolic rate results with the biophysical data from the new DHM assay.

## 2. Materials and Methods

### 2.1. Cell Lines and Cell Culture

Experiments were performed with RAW 264.7 mouse macrophages, NIH-3T3 mouse fibroblasts, NRK-52E rat epithelial kidney cells, and RLE-6TN rat lung epithelial cells. The selection of cell lines consider features and cellular diversity of different organ origins, cell, and uptake types and allows identification of cell type-dependent cytotoxicity [15]. Cell lines were cultured according to the standard cell culture procedures without antibiotics [15]. Mycoplasma contamination was frequently controlled by a commercial qPCR kit (Sartorius, Göttingen, Germany). RAW 264.7 mouse macrophages (ATCC^®^ TIB-71TM, American Type Culture Collection, Manassas, VA, USA) were cultured in Dulbecco’s Modified Eagle Medium (DMEM, Sigma-Aldrich, St. Louis, MO, USA) supplemented with 10% fetal calf serum (FCS, PAN-Biotech, Aidenbach, Germany), 1 mM Pyruvate (Biochrom, Berlin, Germany), and 2 mM Glutamine (Merck, Darmstadt, Germany), passaged twice a week. NIH-3T3 mouse embryonic fibroblasts (ATCC^®^ CRL-1658TM) were cultured in DMEM supplemented with 10% FCS, 1 mM Pyruvate, and 2 mM Glutamine, passaged three times a week. NRK-52E rat kidney epithelial cells (ATCC^®^ CRL-1571TM) were cultured in DMEM supplemented with 10% FCS, 1 mM Pyruvate, and 2 mM Glutamine, passaged twice times a week. RLE-6TN rat lung epithelial cells (ATCC^®^ CRL-2300TM) cultured in Roswell Park Memorial Institute (RPMI, Sigma-Aldrich, St. Louis, MO, USA) medium supplemented with 10% FCS, 1 mM Pyruvate, and 2 mM Glutamine, passaged twice times a week, passages 5-20 were used for DHM QPI cell proliferation experiments and WST-8 cell viability assays.

### 2.2. Nanomaterials

For the study, a set of polymeric and lipid-based nanomaterials were used. LipImage™ 815 lipidots^®^ [36,37,38] for biomedical imaging applications (particle size (DLS): 52.2 nm, polydispersity index: 0.102, endotoxin contamination: < 1 EU/mL) were provided by CEA-Leti (Grenoble, France). These lipid-based particles contained an IR780-lipid dye in their lipophilic core and were synthetized by solvent evaporation method. Empty PACA nanoparticles [39] for drug delivery (particle size (DLS): 134 nm, PDI: 0.11, endotoxin contamination: < 1 EU/mL), and cabazitaxel-loaded PACA nanoparticles [40,41] for cancer treatment (particle size (DLS): 140 nm, PDI: 0.13, endotoxin contamination: <1 EU/mL) were provided by SINTEF Industries (Trondheim, Norway). These PEGylated nanoparticles were synthetized by emulsion polymerization from a water phase containing the alkyl cyanoacrylate monomers and the aqueous phase containing hydrochloric acid and the PEG-surfactants. All nanoparticle dispersions and digitonin solutions (Sigma-Aldrich, St. Louis, MO, USA) were prepared freshly from the manufacturer stocks before each cell culture experiment.

### 2.3. Cell Preparation for Time-Lapse DHM QPI Proliferation Experiments

**Figure 1:**
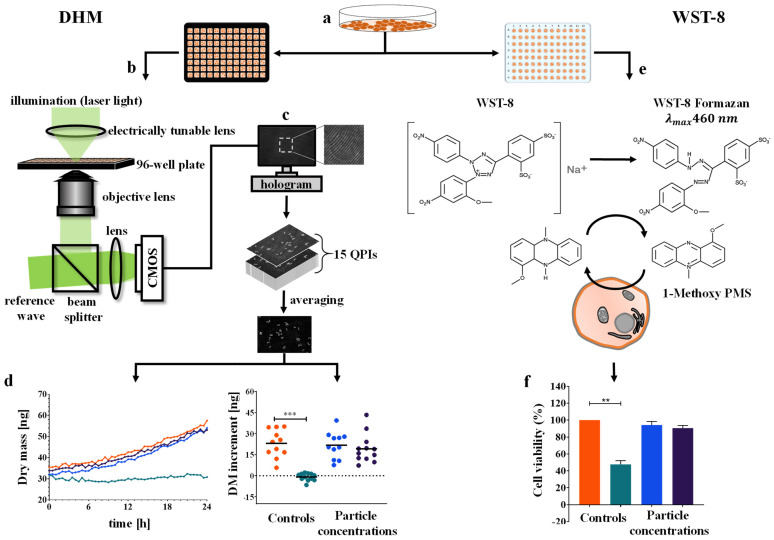
Experimental workflow of the DHM in vitro assay for cytotoxic effect quantification of organic nanoparticles in development for medical use and WST-8 cell viability assay. (**a**) Cell lines were transferred from cell culture vessels to 96-well imaging and assay plates for microscopic and colorimetric viability assays. (**b**) DHM measurements were performed with a modular off-axis setup based on an inverted research microscope and the sample placed inside a stage-top incubator. Sequences of 15 DHM QPI images with modulated object illumination via an electrically tunable lens were acquired immediately after nanomaterial incubation at *n* = 4 measurement positions per well every 30 min for 24 h. (**c**) The QPI image sequences of 15 quantitative phases at each individual measurement point per position were averaged to reduce the impact of coherence-induced image disturbances that are induced by the laser light. (**d**) For each experiment, the temporal dry mass course and the dry mass increments after 24 h in 12 fields of view from three independent experiments were determined from the averaged QPI images, and subsequently, the mean values were calculated. (**e**) Experimental procedure for the WST-8 viability assay. Colorimetric readout of 24 technical repeats total per concentration in three independent experiments was performed after 24 h of incubation with nanoparticles and controls. (**f**) Effects, quantified in three independent experiments, were reported as viability percentages of medium control cells. **, ***: Statistical significances were determined for both assays.

Cells were cultivated for 3–5 days up to a confluence of 90%. Afterward, cells were harvested and seeded into 96-well imaging plates (µ-Plate 96 Well Black, ibidi, Munich, Germany) for DHM QPI experiments (Figure 1a). For this purpose, cells were centrifuged at 330× *g* for 5 min after harvesting with trypsin/EDTA (Sigma Sigma-Aldrich, St. Louis, MO, USA) and transferred into black 96-well imaging plates for DHM QPI experiments. NIH-3T3, NRK-52E, and RLE-6TN cells were seeded at a density of 15,000 cells/well (RAW 264.7 50,000 cells/well) in 300 µL complete cell culture medium. DHM QPI time-lapse experiments were performed on subconfluent cell populations. Well plates were incubated at 37 °C and 5% CO_2_ for 24 h before replacing the medium with complete cell culture medium containing digitonin or nanoparticles in defined concentrations. For DHM experiments, cells were either incubated with medium control, digitonin cytotoxicity control, or nanoparticles, and then placed in the preheated incubator chamber (ibidi, Munich, Germany) of a Nikon Ts2R microscope with attached DHM QPI module as described in section “Time lapse QPI by DHM”. Four positions per well (*n* = 4) were imaged in each of three independently performed experiments (*N* = 3).

### 2.4. Time-Lapse QPI by DHM

An inverted Nikon Ts2R microscope (Nikon, Tokyo, Japan) equipped with an attached DHM module, based on concepts described previously [23], and a motorized microscope stage (Märzhäuser, Wetzlar, Germany) was applied for bright-field imaging and quantitative phase imaging (QPI) of living cells (Figure 1b). A stage top incubator chamber with a heating system (K-frame heating system, ibidi GmbH, Munich, Germany) and gas incubation system (K-frame gas control system, ibidi GmbH, Munich, Germany) allowed time-lapse investigations of living cells in a 5% CO_2_ atmosphere at physiological temperature (37 °C). The coherent light source for the recording of digital holograms was a fiber-coupled solid-state laser (Cobolt 06-DPL, λ = 532 nm, Cobolt AB, Solna, Sweden). Digital off-axis holograms of the specimens were recorded with a complementary metal-oxide-semiconductor (CMOS) sensor (UI-3260CP-M-GL, IDS GmbH, Obersulm, Germany) using a 20x microscope objective (Nikon Plan 20x/0.4, Nikon, Japan) every 30 min for 24 h.

Initially selected field of views (FOVs) at the beginning of the imaging process were observed during the whole experimental period of 24 h. FOVs at t = 0 were selected individually with regard to representative cell confluencies for each cell line and to achieve cell numbers > 100 cells within 12 FOVs for each individual experiment, in accordance with earlier studies on the reproducibility of cell analysis with DHM [42]. For each measurement and time point, one bright-field image and 15 holograms were captured while the object illumination wave was modulated by an electrically tunable lens (ETL) [43]. The reconstruction of quantitative phase images from the digitally captured holograms was performed numerically by a variant of the Fourier transformation method as described before [44], utilizing custom-built software implemented in python 3.7. In short, off-axis holograms were transferred to the spatial frequency domain using a fast Fourier transformation (FFT) where twin image and zero-order intensity contributions, as well as the linear phase shift due to the off-axis tilt, were eliminated. Afterward, an inverse FFT was applied to retrieve the complex object wave, from which subsequently the object wave phase distribution modulo 2π was calculated with the atan2 function. Spherical phase aberrations, such as caused by the microscope objective and ETL, were determined from the size of the frequency sideband of the real image [44] and then subtracted from the initially reconstructed phase map modulo 2π, to achieve a plane phase background without the need of numerical fitting procedures, recording of specimen-free reference holograms or previous knowledge about specimens within the FOV. Optional numerical refocusing was applied, utilizing a convolution approach [24] to propagate the object wave to the image plane if cells were not optically in focus during hologram recording. Subsequently, after reconstruction, unwrapped quantitative phase images for every position and time point were averaged to reduce coherence-induced image disturbances [43]. Irregular spatial phase background fluctuations, such as caused by optical thickness variations of the bottom of the applied polymer well chambers used, were compensated by the ImageJ feature “Subtract background” based on the rolling ball algorithm [45] with identical parameters for all conducted experiments. The resulting averaged and background corrected QPI images contain information about the phase shift Δ*φ*_cell_ induced by the cells as described by Equation (1), where *n*_cell_ is the integral cellular refractive index which is proportional to the concentration of the entire intracellular content, *n*_medium_ is the refractive index of the cell culture medium and *d*_cell_ is the thickness of the cells [46].

(1) $$\Delta \varphi_{\mathrm{cell}}=\frac{2\pi }{\lambda }d_{\mathrm{cell}}\left(n_{\mathrm{cell}}-n_{\mathrm{medium}}\right)$$

Due to the experimental configuration of our study, the absolute parameters *d*_cell_ and *n*_cell_ (Equation (1) are unknown and can change temporally after incubation of cells with nanomaterials, for example, as caused by toxicity-related water content alterations or membrane bursts [40,47]. We thus monitored instead of ∆*φ*_cell_ the temporal dry mass course and the dry mass increment of the entire cell population within the field of view (FOV) after 24 h, which is independent of the cellular water content, as described below.

### 2.5. Determination of the Cellular Dry Mass from Quantitative Phase Images

The temporal dry mass course and the dry mass increment of the entire cell population after 24 h were calculated from the average induced phase shift $\Delta \bar{\varphi }$ (calculated from Equation (1) within the FOV as described with details in [34]:

(2) $$dm=\frac{\lambda }{2\pi \alpha }\Delta \bar{\varphi }S_{\mathrm{FOV}}$$

In Equation (2) $S_{\mathrm{FOV}}$ represents the area of observed field of view = 450 µm × 338 µm = 152,100 µm^2^ while λ = 532 nm represents the light wavelength of the utilized laser. For the specific refractive index increment α, which relates the phase shift to the intracellular protein content, following [48,49,50], the value 0.19 × 10^−3^ mm^3^/g (≙ 0.19 mL/g) was assumed.

### 2.6. WST-8 Cell Viability Assay

A colorimetric WST-8 cell viability assay was performed in parallel to DHM QPI experiments, as illustrated in Figure 1e [51]. Cells harvested with trypsin/EDTA from culture dishes were seeded in a density of 50,000 cells/mL (5000 cells/well) for the cell lines NIH-3T3, NRK-52E, and RLE-6TN and 150,000 cells/mL (15,000 cells/well) for RAW 264.7 in 96-well plates. The assay plates were incubated for 24 h at 37 °C and 5% CO_2_. After an incubation time of 24 h for attachment and differentiation of cells, the medium was replaced with a control agent and nanomaterial containing cell culture medium. Upon exposure of cells with controls and nanoparticles, 96-well plates were incubated for 24 h at 37 °C and 5% CO_2_. Upon completion of incubation time with control agents and nanoparticles, the medium was aspired and replaced by 100 µL warm cell culture medium. After 4 min of equilibration, the medium was removed and 100 µL of freshly prepared, warm WST-8 working medium (0.7 mM WST-8 (2-(2-methoxy-4-nitrophenyl)-3-(4-nitrophenyl)-5-(2,4-disulfophenyl)-2H-tetrazolium, monosodium salt (Carbosynth Ltd., Compton, UK); 0.0134 mM 1 1m-PMS 1-methoxy-5-methylphenazinmethosulfate (Sigma Sigma-Aldrich, St. Louis, MO, USA) in complete cell culture DMEM) were added to all wells. The assay plates were then incubated for 60 min at 37 °C and 5% CO_2_. After the incubation time, the plates were placed in a spectrophotometer and shaken for 2 min. The absorption of the reduced WST-8 formazan was measured at 450 nm (CLARIOstar, BMG Labtech, Ortenberg, Germany), and the reference wavelength 620 nm was subtracted. Medium control was set to 100% viability. Independent experiments were repeated at least 3 times (*N* = 3) with 24 technical replicates total (*n* = 24) of each sample and control. Overall viability percentages the mean and standard deviation were calculated.

### 2.7. Statistical Analysis

All data were produced in three independent experiments (*N* = 3). Statistical significance was calculated using GraphPad Prism version 8.3.0 for the dry mass increment measurements according to double-sided Student’s *t*-test, and significance levels were given as *p* < 0.005(***), *p* < 0.01(**), and *p* < 0.05(*). A pre-test was performed to confirm the applicability of the chosen *t*-test, and Bonferroni correction was applied. An ANOVA was performed for the statistical analysis of the WST-8 assay results, and significance levels were given as *p* < 0.005(***), *p* < 0.01(**), and *p* < 0.05(*).

## 3. Results

### 3.1. Qualitative Analysis of Cell Morphology and Proliferation Alterations in DHM QPI Images after Incubation with Nanoparticles

**Figure 2:**
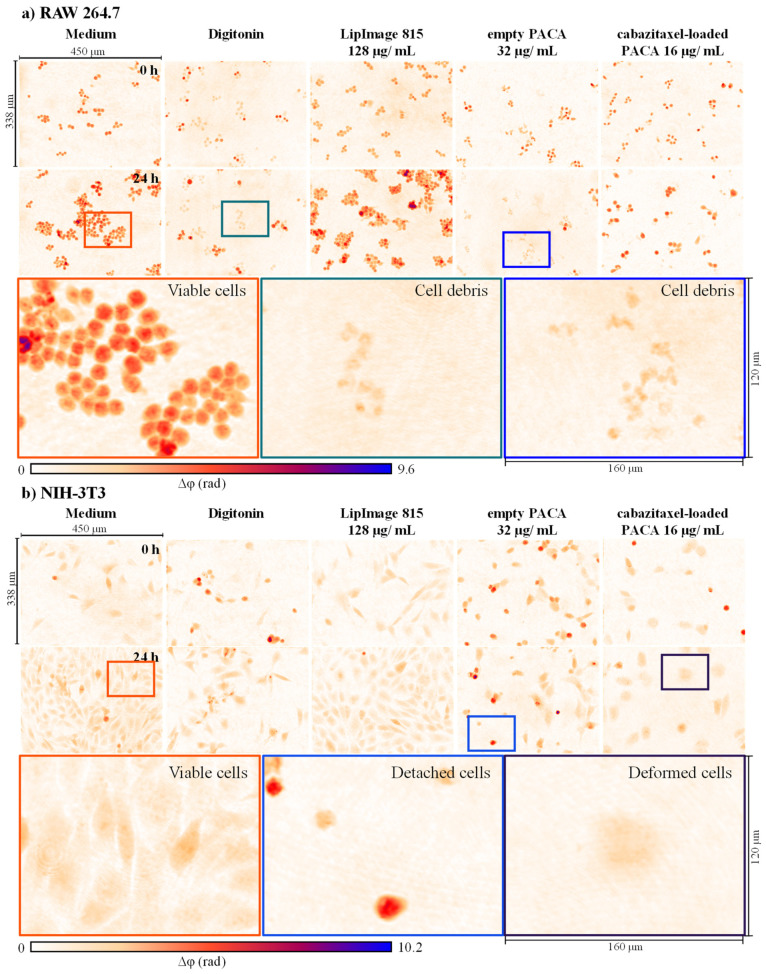
Representative false color-coded DHM QPI images of RAW 264.7 macrophages (**a**) and NIH-3T3 fibroblasts (**b**) incubated with controls and nanoparticles at time points t = 0 h and t = 24 h. For both cell lines, cell culture medium control and cytotoxicity control digitonin is shown. In digitonin controls, cell proliferation was inhibited from time point t = 0 h to t = 24 h. Orange boxes show enlarged areas with viable cells in cell culture medium control. Enlarged areas in green boxes indicate cell debris of lysed RAW 264.7 macrophages. Cells incubated with 128 µg/mL of LipImage™ 815 lipidots^®^ nanoparticles proliferated and grew to a similar extent as observed in the cell culture medium control. For cells incubated with 32 µg/mL of empty PACA nanoparticles, cell proliferation was inhibited. Detached cells are shown in the blue framed enlarged image areas. Incubation with 16 µg/mL of cabazitaxel-loaded PACA nanoparticles caused cell deformation, indicated in the enlarged purple framed area, and similar to PACA nanoparticles, proliferation was inhibited.

**Figure 3:**
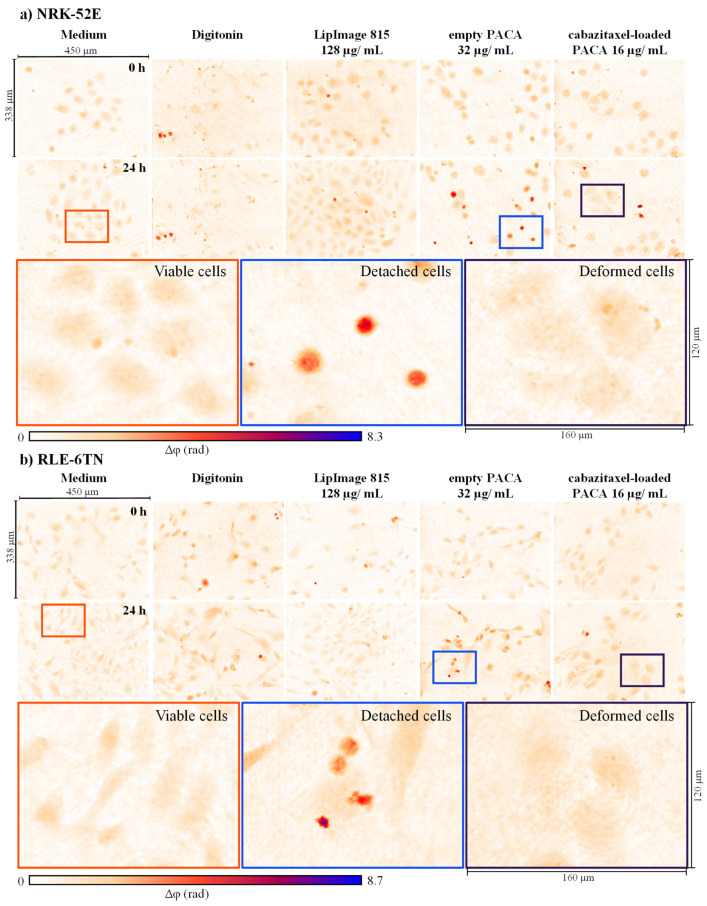
Representative false color-coded DHM QPI images of NRK-52E cells (**a**) and RLE-6TN cells (**b**) incubated with controls and nanoparticles at time points t = 0 h and t = 24 h. For both cell lines, cell culture medium control and cytotoxicity control digitonin is shown. In digitonin controls, cell proliferation was inhibited during the 24 h observation period—enlarged areas framed in orange show viable cells in cell culture medium control. Cells incubated with 128 µg/mL of LipImage™ 815 lipidots^®^ nanoparticles proliferated and grew to a similar extent as observed in the cell culture medium control. Incubation with 32 µg/mL of empty PACA nanoparticles caused inhibited cell proliferation, and detached cells are shown in blue framed enlarged image areas. Incubation with 16 µg/mL of cabazitaxel-loaded PACA nanoparticles caused the deformation of cells as indicated in the purple framed enlarged image area, and proliferation was inhibited.

In an initial step, representative DHM QPI images of cell populations were qualitatively evaluated for cell proliferation and changes in cell morphology. Figure 2 and Figure 3 present false color-coded DHM QPI images with typical proliferation patterns of RAW 264.7 macrophages, NIH-3T3 fibroblasts, as well as NRK-52E kidney and RLE-6TN lung epithelial cells in cell culture medium control, digitonin, and after incubation with nanoparticles at t = 0 h and t = 24 h. For a better visualization, corresponding sets of captured bright-field images for the same FOVs (Figures S2 and S3) and gray level coded versions of Figure 2 and Figure 3 (Figures S4 and S5) are provided in the supplementary material. Supplementary Figure S1 shows gray level coded QPI images of the data in Figure 2 and Figure 3 that are normalized to the phase range of the entire image data set for illustration of the relative thickness differences between the four cell types, respectively. Color-framed enlarged image sections in Figure 2 and Figure 3 indicate representative effects observed in QPI images.

RAW 264.7 macrophages proliferated in clusters in the cell culture medium control (orange box in Figure 2a), while digitonin reduced proliferation and cell debris are visible (green box in Figure 2a). For LipImage™ 815 lipidots^®^ nanoparticles, neither alterations of morphology nor proliferation were apparent compared with the cell culture medium control. Empty PACA nanoparticles severely impacted RAW 264.7 cells. Almost all cells were damaged, and cell debris were visible in DHM QPI images (blue box in Figure 2a). In contrast, macrophages were not lysed by the applied cabazitaxel-loaded PACA nanoparticle concentrations but inhibited in proliferation, and single cells showed an increased phase contrast (Figure 2a).

NIH-3T3 fibroblasts grew to confluent monolayers in cell culture medium control (orange box in Figure 2b) while digitonin reduced proliferation. For LipImage™ 815 lipidots^®^ nanoparticles, no effect compared with the cell culture medium control was observed. Instead, in DHM QPI images of empty PACA nanoparticles, cell detachment was detected (blue box in Figure 2b). Cabazitaxel-loaded PACA nanoparticles reduced proliferation and number of NIH-3T3 cells within the 24-h observation period, and at t = 24 h deformed cells were visible (purple box in Figure 2b).

NRK-52E and RLE-6TN epithelial cells grew towards confluency within the 24-h time frame (orange boxes in Figure 3), while digitonin for both cell types reduced proliferation. Both NRK-52E and RLE-6TN cells, detached upon incubation with empty PACA nanoparticles (blue boxes in Figure 3). Like RAW 264.7 and NIH-3T3, for LipImage™ 815 lipidots^®^ nanoparticles for both epithelial cell types, no effects compared with the cell culture medium control were observed. Empty PACA nanoparticles reduced proliferation and cell number of NRK-52E and RLE-6TN cells and induced cell detachment (blue boxes in Figure 3). Cabazitaxel-loaded PACA nanoparticles reduced proliferation similar to their empty variant but caused cell deformation for both epithelial cell types after 24 h (purple boxes in Figure 3).

The observations in QPI-images in Figure 2 and Figure 3 were also evident in the correlative captured bright-field images (Figures S2 and S3 of Supplementary Materials). In summary, DHM QPI images allowed an initial qualitative analysis for alterations in cell morphology and proliferation to yield the first insights into nanoparticle cytotoxicity and cellular responses.

### 3.2. Time-Resolved Dry Mass Dynamics of Nanoparticle Incubated Cell Populations

**Figure 4:**
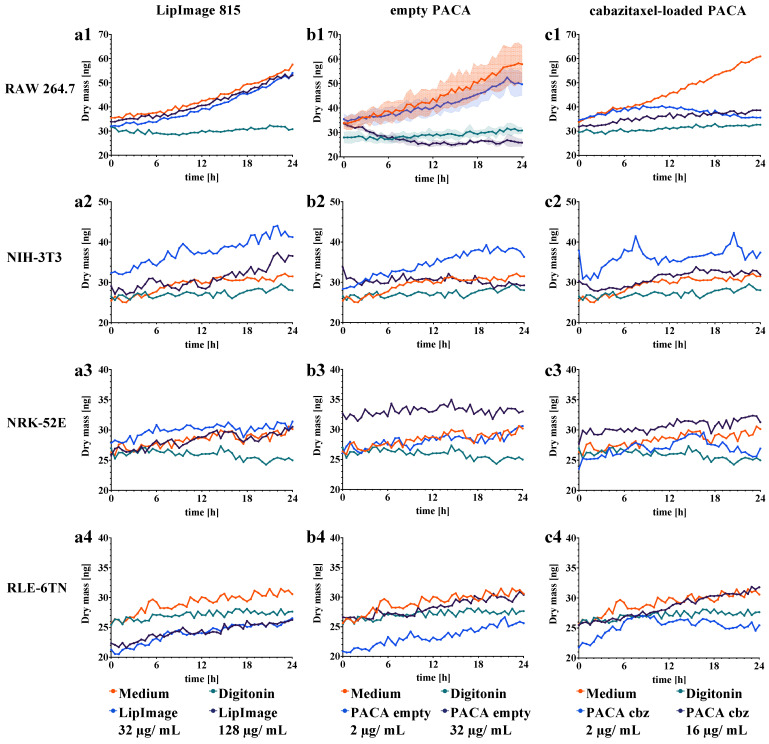
Temporal dry mass development of cell populations in the FOV retrieved from DHM time-lapse measurements for medium control, digitonin cytotoxicity control, and the test set of nanoparticles for RAW 264.7, NIH-3T3, NRK-52E, and RLE-6TN cells. Each data point represents the average dry mass value of *n* = 12 FOVs that were acquired in *N* = 3 independent experiments (*n* = 4 FOVs per independent experiment). Column (**a1**–**a4**): Cell lines after incubation with medium and digitonin cytotoxicity controls, 32 µg/mL and 128 µg/mL of LipImage™ 815 lipidots^®^ nanoparticles. Column (**b1**–**b4**): Cell lines after incubation with controls and 2 µg/mL and 32 µg/mL of empty PACA nanoparticles. Column (**c1**–**c4**): Cell lines after incubation with controls and 2 µg/mL and 16 µg/mL of cabazitaxel-loaded PACA nanoparticles. To illustrate the variation during the experimental period, in b1, representative standard deviations are plotted for empty PACA incubated RAW 264.7 macrophages. For clarity, the standard deviation is not shown for the other dry mass developments. Figure S6 in the supplement shows all plots with standard deviations included.

To gain insights into the temporal response of cell proliferation after incubation with nanoparticles, the dry mass development of cell populations in the FOV was quantified in comparison to the cell culture medium and digitonin cytotoxicity controls. Cell culture medium and digitonin controls were either repeated individually for each tested nanoparticle (RAW 264.7) or included within a measurement consisting of all three tested nanoparticles in a single DHM experiment (NIH-3T3, NRK-52E, RLE-6TN). Figure 4 shows plots of the dry mass dynamics for all investigated cell lines after incubation with the different nanomaterials. Each data point represents the cell mean dry mass amount determined from measurements in *n* = 12 individually continuously observed FOVs that were acquired at different sample areas during three independent experiments (*N* = 3). To illustrate typical variations of the dry mass data during the experimental period, representative standard deviations for empty PACA incubated RAW 264.7 macrophages were plotted (Figure 4(b1)), while for clarity of presentation in all other plots, the standard deviation is omitted. Supplementary Figure S6 represents a version of Figure 4 with all values and standard deviations included. In Supplementary Figure S7, a comparative plot of the temporal dry mass development for the cell culture medium control is shown. The plots in Supplementary Figure S7 illustrate that similar values and standard deviations for the dry mass data of all four tested cell lines in *N* = 3 independent experiments were achieved.

For all cell lines in the cell culture medium, a continuous dry mass increase was observed over the observation period of 24 h. While RAW 264.7 displayed a distinct exponential dry mass increase (Figure 4(a1–c1)), the proliferation of the other three cell lines was linear (NIH-3T3: Figure 4(a2–c2), NRK-52E: Figure 4(a3–c3), RLE-6TN: Figure 4(a4–c4)). Cytotoxic effects of the nanoparticles on RAW 264.7 macrophages were evident from the cell dry mass dynamics for all plots in Figure 4. The dry mass graph of RAW 264.7 macrophages for 2 µg/mL of cabazitaxel-loaded PACA nanoparticles increased during the first 10 experimental hours but decreased to digitonin control levels again until 24 h (Figure 4(c1)). In addition, changes in dry mass dynamics for NIH-3T3, NRK-52E, and RLE-6TN were not as clearly observable as for RAW 264.7 due to a lower signal-to-noise ratio and higher standard deviations of the measurement data (Supplementary Figure S6) and cell migration.

In summary, cell population dry mass dynamics could be extracted from the time-resolved QPI image stacks generated by the DHM assay for nanoparticle cytotoxicity quantification. Dry mass dynamics indicate evident responses in RAW 264.7 macrophages, while impacts on the other three cell lines were less obvious from the graphs of Figure 4.

### 3.3. Dry Mass Increment as Endpoint for Cell Proliferation and Nanoparticles Cytotoxicity

**Figure 5:**
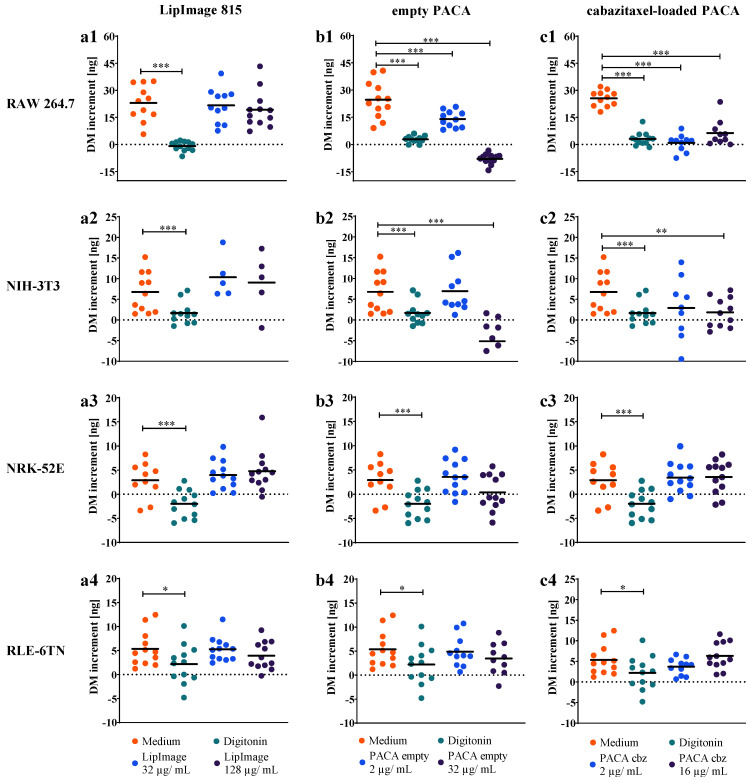
Increment of the cell population dry mass (DM) in the FOV after 24 h of incubation with cell culture medium, digitonin, and nanoparticles. Data points represent the dry mass increment of the cell populations within a single FOV. Data were acquired from different sample areas in 12 FOVs during *N* = 3 independent experiments. LipImage™ 815 lipidots^®^ nanoparticles, (**a1**–**a4**), empty PACA nanoparticles (**b1**–**b4**), and cabazitaxel-loaded PACA nanoparticles (**c1**–**c4**) were tested on RAW 264.7, NIH-3T3, NRK-52E, and RLE-6TN cells. Each experiment included a set of corresponding controls: cell viability medium control and cytotoxicity control digitonin. Significance levels: *p* < 0.005(***), *p* < 0.01(**), *p* < 0.05(*).

To investigate if QPI with DHM is capable of generating endpoint data for quantification of proliferation and of identifying statistically significant nanoparticle effects, from the data in Figure 4, the cell dry mass increments after 24 h with respect to t = 0 h were calculated. Therefore, the dry mass of the cell population in the individual FOVs at t = 0 h was subtracted from the values measured at t = 24 h, as described in the method section. Figure 5 shows the resulting scatterplots of the cell population dry mass for treatments with medium control, digitonin cytotoxicity control, and nanoparticles. The numbered rows (1–4) of Figure 5 indicate the results for the four different cell lines included in the study, while the columns (a–c) show the corresponding data obtained from each cells line after incubation with the three different nanoparticles.

The dry mass increment of RAW 264.7 macrophages (Figure 5(a1–c1)) was measured in the range from 23.0 ng and 25.5 ng per FOV under cell culture medium control conditions in all nanoparticle experiments performed. Dry mass of NIH-3T3 (Figure 5(a2–c2)), NRK-52E (Figure 5(a3–c3)), and RLE-6TN (Figure 5(a4–c4)) cells increased between 2.9 ng and 6.8 ng in the observed time frame in each experiment performed with the nanoparticles. The cytotoxicity control digitonin inhibited dry mass increment in all four cell lines between −2.0 ng and 3.1 ng in the 24-hour period in all experiments performed with the nanoparticles (all plots in Figure 5). This was observed in all individual digitonin incubation experiments and significant in all cell lines (all *p* < 0.005; except RLE-6TN *p* < 0.01).

We did not detect any effect on cell dry mass in the DHM assay for the LipImage™ 815 lipidots^®^ on the four cell lines in two applied concentrations 32 µg/mL and 128 µg/mL as shown in Figure 5(a1–a4). Dry mass increments quantified in the DHM assay after 24 h did not differ from the medium control (Figure 5(a1–a4)).

Empty PACA nanoparticles affected the 24-h dry mass increment of the four cell lines to a different extent; RAW 264.7 macrophages were affected by 2 µg/mL and dry mass only increased by 14.0 ± 4.5 ng in the observed time frame (*p* < 0.005), while 32 µg/mL caused a negative dry mass increment of −7.9 ± 2.9 ng due to cell lysis (Figure 5(b1)). NIH-3T3 fibroblasts were significantly affected by 32 µg/mL of empty PACA nanoparticles (*p* < 0.005), and a mean negative dry mass increment of −5.1 ± 7.5 ng was observed, as shown in Figure 5(b2). RLE-6TN lung epithelial cells and NRK-52E kidney epithelial cells did not respond to the incubation of 2 µg/mL and 32 µg/mL of empty PACA nanoparticles, dry mass development over the 24-hour period was not reduced, and dry mass increments after 24 h did not significantly differ from the medium control cell populations as shown in Figure 5(b3,b4).

For the cabazitaxel-loaded PACA nanoparticles, we observed cytotoxic effects on RAW 264.7 macrophages and NIH-3T3 fibroblasts. RAW 264.7 macrophages showed reduction in dry mass increments to 0.9 ± 4.7 ng (*p* < 0.005) and 6.3 ± 7.1 ng (*p* < 0.005) for 2 and 16 µg/mL of cabazitaxel-loaded PACA nanoparticles quantified by DHM over 24 h (Figure 5(c1)). The fibroblast cell line NIH-3T3 was significantly affected by 16 µg/mL of cabazitaxel-loaded PACA nanoparticles with a reduction in 24-h dry mass increment to 2.6 ± 1.4 ng (*p* < 0.01) per cell population in one FOV (Figure 5(c2)).

We summarize that the calculations of 24-h dry mass increments retrieved from DHM QPI images allowed an endpoint quantification of proliferation and nanoparticle cytotoxicity. Moreover, we could observe not only different proliferation rates in the cell culture medium control for the cell types but also extract absolute biophysical proliferation data at ng dry mass scale. In addition to the time-resolved dry mass dynamics of Figure 4, the calculation of 24-h dry mass increments allowed the identification of significant cell responses to nanoparticle exposure.

### 3.4. WST-8 Cell Viability Assay

**Figure 6:**
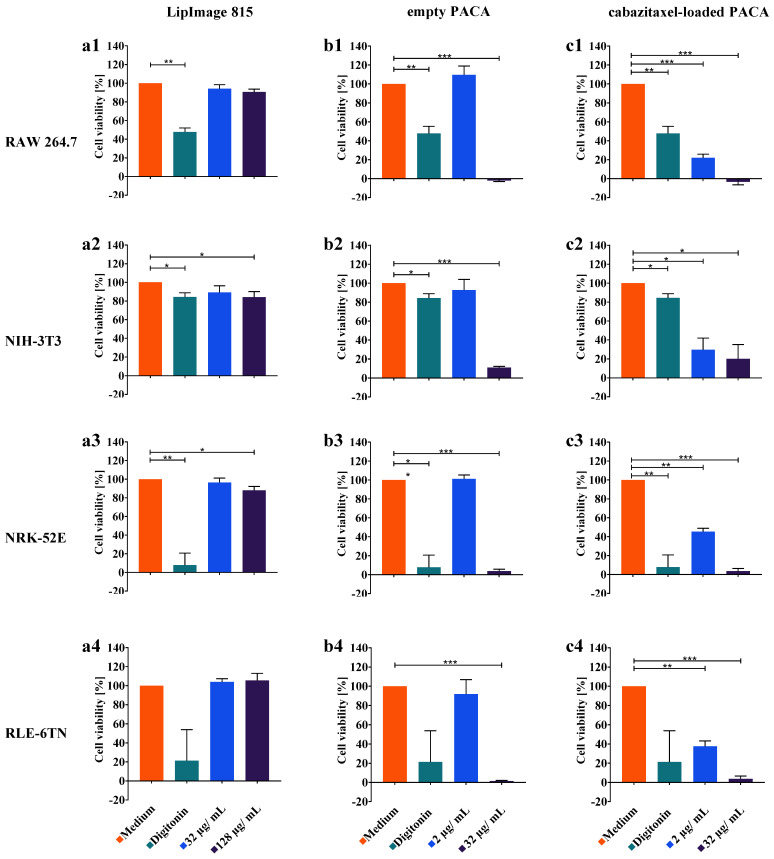
Cell viability of the four cell lines incubated with nanoparticles and controls measured by the colorimetric WST-8 assay. Cell culture and cell seeding procedures were performed in analogy to DHM QPI experiments, except for cabazitaxel-loaded PACA nanoparticles, where the concentration was increased to 32 µg/mL with respect to comparability to their empty counterpart (for further details, see text). Photometric readout of assay plates was performed 24 h after incubation of cells with controls and nanomaterials. Medium control served as a reference point for cell viability and was set to 100%. LipImage™ 815 lipidots^®^ (**a1**–**a4**), empty PACA nanoparticles (**b1**–**b4**), and cabazitaxel-loaded PACA nanoparticles (**c1**–**c4**) were tested on the four selected cell lines. Plotted are mean values and standard deviations of 24 technical repeats (*n* = 24) from three independent experiments (*N* = 3). An ANOVA was performed for the statistical analysis of the WST-8 assay results, and significance levels were given as *p* < 0.005(***), *p* < 0.01(**), and *p* < 0.05(*).

In parallel to the investigations with DHM, a colorimetric WST-8 cell viability assay was performed as described in the methods section. Different from the DHM assay, in which *n* = 12 FOVs were observed during *N* = 3 independent experiments, the WST-8 assay was performed in *n* = 24 wells within *N* = 3 independent technical repeats. Moreover, in contrast to the DHM assay, with respect to comparability with their empty counterpart, the concentration of cabazitaxel-loaded PACA nanoparticles was increased to 32 µg/mL. The concentration of 32 µg/mL of cabazitaxel-loaded PACA nanoparticles was not applicable for DHM measurements as it immediately caused lysis of most cells for all cell types, preventing QPI time-lapse observations. All other WST-8 assay parameters, including incubation times, cell densities and handling, nanoparticle batches, and concentrations, were adjusted equally to the DHM assay. Cell viability data for digitonin and the nanoparticles were normalized to the cell culture viability medium control and are presented as percent of the medium control in Figure 6. The columns a–c of Figure 6 summarize the results of the WST-8 assay for the three nanoparticles achieved with respect to the different cell lines in corresponding rows 1–4.

Similar to the dry mass developments and increments presented above, in Figure 4 and Figure 5 the metabolic rate quantified in the WST-8 assay was reduced up to 80% by the cytotoxicity control digitonin in all nanoparticle exposure experiments for all cell lines (RAW 264.7: Figure 6(a1–c1), NRK-52E: Figure 6(a3–c3), and RLE-6TN: Figure 6(a4–c4)), while for NIH-3T3 (Figure 6(a2–c2)) the lowest response was observed.

As quantified in the DHM assay, the WST-8 assay showed no significant reduction in cell viability for the LipImage™ 815 lipidots^®^ nanoparticles, except for NIH-3T3 fibroblasts at 128 µg/mL (Figure 6(a2)) and NRK-52E kidney epithelial cells (Figure 6(a3)). The data suggest a similar response of the cell line proliferation as quantified by DHM, and in the metabolic rate detected by WST-8 assay when incubated with LipImage™ 815 lipidots^®^ nanoparticles. Albeit, small effects on the metabolic rate were observed in NIH-3T3 and NRK-52E at 128 µg/mL, for which the DHM assay did not show a reduction in cell proliferation.

For the empty PACA nanoparticles, the colorimetric WST-8 assay showed high effects for the RAW 264.7 macrophages at a concentration of 32 µg/mL, while 2 µg/mL had no effect on cell viability (Figure 6(b1)). WST-8 and DHM assay findings align well for this particle type. While no significant changes in dry mass development for two epithelial cell lines NRK-52E and RLE-6TN were present, the WST-8 assay detected significantly reduced metabolic activity for 32 µg/mL (Figure 6(b3,b4)). NIH-3T3 fibroblasts showed a significant effect (*p* < 0.05) at both applied concentrations in the WST-8 assay (Figure 6(b2)).

A reduction in metabolic rate for the cabazitaxel-loaded PACA nanoparticles quantified in the WST-8 assay was detected beginning from a concentration of 2 µg/mL and decreased to 0% at 32 µg/mL in all four cell lines (Figure 6(c1–c4)). The reduction in cell viability percentages was higher in the cabazitaxel-loaded variant of the PACA nanoparticles compared to the empty variant, and both applied concentrations caused significant effects in the four cell lines (Figure 6(c1–c4)).

In summary, most effects of nanoparticles on cell viability measured with WST-8 assay corresponded well with the results in Figure 2, Figure 3, Figure 4 and Figure 5 for the developed DHM assay. Differences were observed for the two epithelial cell lines (RLE-6TN, NRK-52E), which responded more sensitively in the WST-8 assay to PACA nanomaterials, and for the LipImage™ 815 lipidots^®^ nanoparticles where a slight effect on WST-8 assay was detected for NIH-3T3 and NRK-52E.

## 4. Discussion

In our study, a DHM-based assay for cytotoxicity quantification was evaluated using a set of three organic nanoparticles and four cell lines. The objective was to investigate the capabilities of this QPI method to extend the available nanomaterial in vitro toxicity assays. A standardized colorimetric WST-8 cell viability assay was performed in parallel to gain a better understanding of the advantages and limitations of the label-free DHM assay. Figure 1 illustrates the experimental workflow of the two methods. A custom build DHM system with a motorized stage and stage-top incubation unit, as described before, was adapted for nanoparticle testing [23]. From the holograms recorded in the DHM-based assay, QPI images were reconstructed [44] and evaluated qualitatively for effects of the nanoparticles on cell morphology and proliferation, as shown in Figure 2 and Figure 3. Subsequently, cell dry mass dynamics over 24 h and dry mass increments per FOV were calculated as a measure for proliferation and cytotoxicity effects as described in the methods section.

LipImage™ 815 lipidots^®^ are lipid nanocarriers developed for in vivo fluorescence bioimaging and are described in the literature as non-cytotoxic [37]. We confirmed the non-toxic nature of LipImage™ 815 lipidots^®^ nanoparticles by imaging all four tested cell lines in the quantitative and label-free optical DHM assay and colorimetric WST-8 viability assay. The QPI images in Figure 2 and Figure 3 showed no evident alterations in cellular morphology and proliferation. Moreover, no effects were quantified in the cell population dry mass dynamics or increments (Figure 4(a1–a4) and Figure 5(a1–a4)). The colorimetric WST-8 cell viability assay also detected no effect of LipImage™ 815 lipidots^®^ on cell viability, but at the concentration of 128 µg/mL, a slight significant reduction in cell viability to 80% and 83% (both *p* < 0.05) were measured for NIH-3T3 fibroblasts and NRK-52E kidney epithelial cells, respectively (Figure 6(a2,a3)). These findings are in accordance with published WST-1 assay data for NIH-3T3 fibroblasts [37]. For the non-toxic LipImage™ 815 lipidots^®^ the measured endpoint of the two assays was evaluated as equal.

PACA nanoparticles are frequently used in pharmaceutical technology as nanocarriers for contrast agents and cytostatic drugs with the benefit of increasing active pharmaceutical ingredient concentrations at the site of interest, e.g., solid tumor tissue [39,40]. In contrast to the LipImage™ 815 lipidots^®^, the empty PACA nanoparticles showed a more differential pattern in DHM and WST-8 assays: cell dry mass increments of the epithelial cells NRK-52E and RLE-6TN were not affected (Figure 5(b3,b4)), while a reduced metabolic rate in the two cell lines was detected in the WST-8 assay at both applied particle concentrations of 2 µg/mL and 32 µg/mL (Figure 6(b3,b4)). QPI images of NRK-52E and RLE-6TN showed slight alterations in morphology (Figure 3a,b). For this combination of nanoparticles and cells, the colorimetric WST-8 assay showed higher sensitivity. DHM detects protein content, whereas the WST-8 assay is based on cell activity, which may be affected at a lower concentration than the DHM parameters. Further determining factors for the lower sensitivity of DHM assay may be a flat cell morphology, slower proliferation rates, and lower signal-to-noise ratios in the epithelial cell lines (Figure S1(a3,a4)). From these findings, we can conclude that careful selection of cell types is important not only due to physiology but also to achieve an adequate QPI image quality. Data obtained for RAW 264.7 macrophages in both DHM and WST-8 assays aligned well: concentrations that reduced the metabolic rate also significantly impacted the dry mass increment (Figure 5(b1,c1) and Figure 6(b1,c1)). The dry mass development of NIH-3T3 fibroblasts was reduced by higher concentrations of empty PACA nanoparticles; likewise, the WST-8 assay detected a significant effect on cell viability. The effects of empty PACA nanoparticles observed in our study are in line with published IC_50_ values for these nanocarriers in various cell lines ranging from 18 µg/mL (OVCAR-3 cells) to over 300 µg/mL (DU-145 cells) [40].

The cabazitaxel-loaded PACA nanoparticles showed a different cytotoxicity profile compared to their empty counterpart, which was evident from the data generated in the DHM assay. NIH-3T3 fibroblasts dry mass increment was significantly affected by 16 µg/mL of cabazitaxel-loaded PACA nanoparticles at 1.8 ± 3.6 ng (*p* < 0.01), while 2 µg/mL of cabazitaxel-loaded nanoparticles had no significant effect at 2.9 ± 7.3 ng (*p* > 0.05) (Figure 5(c2)). The elongated morphology of the fibroblasts was altered by the microtubule-function inhibitor cabazitaxel, visible in the quantitative phase images (Figure 2b). NRK-52E and RLE-6TN cell’s metabolic rate was least affected by the cabazitaxel-loaded PACA nanoparticles (Figure 6(c3,c4)), and dry mass increment after 24 h was also not altered compared to the medium control (Figure 5(c3,c4)). As demonstrated in Figure 4(b1,c2), the kinetics of the induced toxicity in RAW 264.7 macrophages could be observed in the QPI DHM assay, while the readout of the WST-8 assay only yields information for the endpoint of the incubation time after 24 h. For the PACA nanoparticles, it was demonstrated before that the degradation product of these polymeric nanoparticles causes cell damage. A study demonstrated that PACA nanoparticles with fast degradation rates caused more acute cytotoxicity [40]. Effects on cells that occur after a certain incubation time may be linked to the release of cytostatic drugs from the nanocarriers. In addition to the absolute biophysical data of cellular proliferation, the quantitative phase images produced by DHM allowed the time-resolved morphological evaluation of cells.

As proposed by earlier research [14,29,30,52], the data from our study suggests that DHM as a label-free quantitative imaging tool is not only suitable for in vitro cytotoxicity studies but with the promising capability to be applied in the field of medical nanotechnology. In Figure 4, we quantified the time-resolved dry mass development of cell populations, which is an advantage of the minimally invasive and non-phototoxic DHM method. Dry mass increments over 24 h were calculated and shown in Figure 5. They serve as a measure for proliferation and induced cytotoxicity over the 24-h time frame. The impact of baseline phase shift artifacts, as can be induced by the stage-top incubation chamber, are also reduced by these increment calculations. DHM has already proven applicability for engineered nanomaterials, and this study increases the spectrum of applications to organic nanoparticles. The recent developments in QPI have brought the method from a technology research state towards an application-focused stage, which may add value also to other fields like medical nanotechnology. Detection of early cell death, red blood cell aging, and drug discovery research has been performed with available QPI systems [53,54]. In these studies, it was highlighted that the non-invasiveness of DHM and the possibility to reuse biological specimens after DHM imaging is a major benefit of the label-free method. The results from this study on nanoparticle cellular effects quantified by QPI with DHM are in line with these earlier findings. In addition to the quantification of the dry mass developments of entire cell populations within the microscopic FOV as a marker for proliferation, a further significant benefit would be the extraction of single-cell related data from QPI images, such as cell number as well as shape, morphology and motility parameters [55,56]. However, in our study, the limited signal-to-noise ratio achieved from the very thin growing epithelial cell lines NRK-52E and RLE-6TN (see Figure 3 and Supplementary Figures S1, S6 and S7) prevented an adequate image segmentation with standard image processing such as thresholding to extract accurate single cell-related parameters in an automated manner. Moreover, investigated RAW 264.7 macrophages (Figure 2a), although causing high contrast in DHM QPI images, grew with tight cell-cell contacts in clusters, which hindered a reliable threshold-based image evaluation for single-cell data. Here, sophisticated algorithms that, e.g., rely on convolutional neural networks (CNNs) [57] prospect improved biophysical parameter extraction, suitable for subsequent advanced analysis with machine learning approaches [58,59]. This can be used for the detection of subpopulations or phenotypes.

The absence of chemical marker agents that may interfere with nanoparticles makes DHM suitable for more robust applications in medical nanotechnology. The reliability and robustness of DHM assay are demonstrated by the high reproducibility of the data from the temporal dry mass courses of *N* = 3 independent experiments for RAW 264.7 macrophages in medium and digitonin controls (Figure 4(a1–c1)). These observations are in agreement with Supplementary Figure S7 for the cell culture medium controls where similar values and standard deviations for the dry mass development of the four diverse cell lines with different morphological properties (Figure 2 and Figure 3) were achieved. However, slightly higher temporal fluctuations of the standard deviations for NIH-3T3, NRK-52E, and RLE-6TN cells with respect to RAW 264.7 may be caused by higher motilities of these cell types compared with the macrophages and the lower signal-to-noise ratio (Figure S6). These effects may also explain the higher temporal fluctuation of the average dry mass values for those cell lines in Figure 4. The DHM setup used for this study has favorable technical features that make it available for standard biomedical laboratories. QPI with DHM is based on the detection of optical path length changes in transmission which only requires low light intensities for object illumination and thus minimizes the interaction with the sample. This concept enables minimally invasive long-term, time-lapse holographic imaging for quantitative monitoring of dynamic changes of cell morphology, motility, and proliferation by absolute biophysical parameters, such as the cellular dry mass, without the need for previously defined endpoints. Hologram acquisition times in millisecond range with repetition rates up to video frequency in combination with software for automated data acquisition and data retrieval from entire cell populations in the FOV make the interferometry-based technology robust, automatable, and applicable to the biomedical laboratory environment. The measurement of a 96-well plate can be achieved with the current experimental DHM configuration in less than 30 min and further shortened by technical optimization. The efforts for data extraction and evaluation depend on the chosen parameters. In our study, sample preparation for DHM analysis was less laborious compared with the correlative applied WST-8 assay, as no staining steps were required. Moreover, due to the label-free imaging DHM concept, the analyzed cell cultures remain in a native state, which makes the samples available for further downstream analysis. The use of DHM for in vitro toxicity assays is comparable to the setup and use of ELISA readers for WST-8 test readout and requires no special expertise in optical technology. The analysis of the resulting data can be standardized and successfully performed by any staff with experience in biomedical assay analysis. The time-resolved measurements were performed in a simple stage-top incubator without an anti-vibration isolated optical table. The stage-top incubator allows easy to operate sample handling even during measurements in contrast to other DHM systems, which are placed entirely in a cell culture incubator [25]. As described in the Results section, DHM concepts as used in this study may have limitations in detecting cytotoxicity for cells with thin morphology and very slow proliferation rates. This highlights the necessity to carefully select the cellular model used for QPI cytotoxicity studies. For PACA nanoparticles, cell type-dependent cytotoxicity was demonstrated before [40]. Here, we confirmed cell-type dependent effects of organic nanoparticles that are in development for medical use, as well as the importance of the cellular model for the DHM assay. Overall, the DHM assay developed for nanoparticle testing demonstrated to be a valuable extension to the available methods for cytotoxicity testing due to the circumvention interferences with assay chemicals, additional biophysical readout, increased automation, and less laboratory work.

## 5. Conclusions and Future Prospects

In summary, the DHM-based in vitro assay for cytotoxicity assessment of organic nanoparticles in development for medical use is a functional label-free tool for the quantification of cellular proliferation and nanoparticle effects on cell viability. LipImage™ 815 lipidots^®^ for biomedical imaging were confirmed to be non-toxic by the DHM assay in the applied concentrations, while both empty and cabazitaxel-loaded PACA nanoparticles significantly affected cell dry mass development overtime in two of the four cell lines. The parallel performed colorimetric WST-8 cell viability assay supports the results of the quantitative phase microscopy study with the set of organic nanoparticles. DHM demonstrates its potency as a method for preclinical high-throughput assessment of novel agents in nanomedicine by fast and easy-to-use label-free optical quantification of cell proliferation in standard 96-well plates. Furthermore, both assays, DHM and WST-8, could potentially be performed from the same well plates, where WST-8 assay is conducted after the DHM QPI readout. The possibility to combine DHM with other analytical methods is highly valuable to achieve increased data quality in in vitro cytotoxicity experiments. Supernatant and cells of DHM experiments may also be used for downstream biochemical analysis. The added benefit of the DHM QPI assay presented here is the extraction of biophysical parameters in a time-resolved manner in addition to colorimetric endpoint determinations of widely used biochemical assays. In addition to the dry mass calculations on a cell population demonstrated here, the image stacks could be analyzed down to the single-cell level by improved image analysis software. This would further increase the resolution of the generated cytotoxicity data. With the possibilities to incorporate DHM into commercially available microscopes and integration of the method into existing workflows for nanomaterial testing, the presented DHM QPI assay is a highly useful tool for cytotoxicity assessment of nanoparticles and potentially their preclinical development.

## Data Availability

Requests for data and materials should be addressed to the corresponding authors.

## Supplementary Materials

The following are available online at https://www.mdpi.com/article/10.3390/cells11040644/s1, Figure S1: Representative DHM QPI images of the four cell lines; Figure S2: Representative bright-field images of RAW 264.7 macrophages and NIH-3T3 fibroblasts; Figure S3: Representative bright-field images of NRK-52E cells and RLE-6TN cells; Figure S4: Representative DHM QPI images in gray level representation of RAW 264.7 macrophages and NIH-3T3 fibroblasts; Figure S5: Representative DHM QPI images in gray level representation of NRK-52E cells and RLE-6TN cells; Figure S6: Temporal dry mass development of cell populations with standard deviations; Figure S7: Temporal dry mass development of cell populations with standard deviation for cell culture medium control for RAW 264.7, NIH 3T3, NRK 52E, and RLE 6TN cells.

## References

[1] Sadrieh N., Tyner K.M. (2010). Nanotechnology and therapeutic delivery: A drug regulation perspective. Ther. Deliv..

[2] Chang E.H., Harford J.B., Eaton M.A., Boisseau P.M., Dube A., Hayeshi R., Swai H., Lee D.S. (2015). Nanomedicine: Past, present and future—A global perspective. Biochem. Biophys. Res. Commun..

[3] Etheridge M.L., Campbell S.A., Erdman A.G., Haynes C.L., Wolf S.M., McCullough J. (2013). The big picture on nanomedicine: The state of investigational and approved nanomedicine products. Nanomedicine.

[4] Cheheltani R., Ezzibdeh R.M., Chhour P., Pulaparthi K., Kim J., Jurcova M., Hsu J.C., Blundell C., Litt H.I., Ferrari V.A. (2016). Tunable, biodegradable gold nanoparticles as contrast agents for computed tomography and photoacoustic imaging. Biomaterials.

[5] Jeon M., Halbert M.V., Stephen Z.R., Zhang M. (2021). Iron Oxide Nanoparticles as T1 Contrast Agents for Magnetic Resonance Imaging: Fundamentals, Challenges, Applications, and Prospectives. Adv. Mater..

[6] Poon W., Kingston B.R., Ouyang B., Ngo W., Chan W.C.W. (2020). A framework for designing delivery systems. Nat. Nanotechnol..

[7] Malam Y., Loizidou M., Seifalian A.M. (2009). Liposomes and nanoparticles: Nanosized vehicles for drug delivery in cancer. Trends Pharmacol. Sci..

[8] Fries C.N., Curvino E.J., Chen J.-L., Permar S.R., Fouda G.G., Collier J.H. (2020). Advances in nanomaterial vaccine strategies to address infectious diseases impacting global health. Nat. Nanotechnol..

[9] Shin M.D., Shukla S., Chung Y.H., Beiss V., Chan S.K., Ortega-Rivera O.A., Wirth D.M., Chen A., Sack M., Pokorski J.K. (2020). COVID-19 vaccine development and a potential nanomaterial path forward. Nat. Nanotechnol..

[10] Lewinski N., Colvin V., Drezek R. (2008). Cytotoxicity of nanoparticles. Small.

[11] Arora S., Rajwade J.M., Paknikar K.M. (2012). Nanotoxicology and in vitro studies: The need of the hour. Toxicol. Appl. Pharmacol..

[12] Andraos C., Yu I.J., Gulumian M. (2020). Interference: A Much-Neglected Aspect in High-Throughput Screening of Nanoparticles. Int. J. Toxicol..

[13] Piret J.-P., Bondarenko O.M., Boyles M.S.P., Himly M., Ribeiro A.R., Benetti F., Smal C., Lima B., Potthoff A., Simion M. (2017). Pan-European inter-laboratory studies on a panel of in vitro cytotoxicity and pro-inflammation assays for nanoparticles. Arch. Toxicol..

[14] Mues S., Ketelhut S., Kemper B., Schnekenburger J. (2017). Digital Holographic Microscopy as Multimodal Read Out for In Vitro Nanomaterial Cytotoxicity Testing.

[15] Kroll A., Dierker C., Rommel C., Hahn D., Wohlleben W., Schulze-Isfort C., Göbbert C., Voetz M., Hardinghaus F., Schnekenburger J. (2011). Cytotoxicity screening of 23 engineered nanomaterials using a test matrix of ten cell lines and three different assays. Part. Fibre Toxicol..

[16] Bohmer N., Rippl A., May S., Walter A., Heo M.B., Kwak M., Roesslein M., Song N.W., Wick P., Hirsch C. (2018). Interference of engineered nanomaterials in flow cytometry: A case study. Colloids Surf. B Biointerfaces.

[17] Sohaebuddin S.K., Thevenot P.T., Baker D., Eaton J.W., Tang L. (2010). Nanomaterial cytotoxicity is composition, size, and cell type dependent. Part. Fibre Toxicol..

[18] Kroll A., Pillukat M.H., Hahn D., Schnekenburger J. (2009). Current in vitro methods in nanoparticle risk assessment: Limitations and challenges. Eur. J. Pharm. Biopharm..

[19] Bremer-Hoffmann S., Halamoda-Kenzaoui B., Borgos S.E. (2018). Identification of regulatory needs for nanomedicines. J. Interdiscip. Nanomed..

[20] Halamoda-Kenzaoui B., Holzwarth U., Roebben G., Bogni A., Bremer-Hoffmann S. (2019). Mapping of the available standards against the regulatory needs for nanomedicines. WIREs Nanomed. Nanobiotechnol..

[21] Park Y., Depeursinge C., Popescu G. (2018). Quantitative phase imaging in biomedicine. Nat. Photonics.

[22] Lee K., Kim K., Jung J., Heo J., Cho S., Lee S., Chang G., Jo Y., Park H., Park Y. (2013). Quantitative phase imaging techniques for the study of cell pathophysiology: From principles to applications. Sensors.

[23] Lenz P., Bruckner M., Ketelhut S., Heidemann J., Kemper B., Bettenworth D. (2016). Multimodal Quantitative Phase Imaging with Digital Holographic Microscopy Accurately Assesses Intestinal Inflammation and Epithelial Wound Healing. J. Vis. Exp..

[24] Kemper B., Carl D., Schnekenburger J., Bredebusch I., Schäfer M., Domschke W., von Bally G. (2006). Investigation of living pancreas tumor cells by digital holographic microscopy. J. Biomed. Opt..

[25] Kamlund S., Strand D., Janicke B., Alm K., Oredsson S. (2017). Influence of salinomycin treatment on division and movement of individual cancer cells cultured in normoxia or hypoxia evaluated with time-lapse digital holographic microscopy. Cell Cycle.

[26] Huang D., Leslie K.A., Guest D., Yeshcheulova O., Roy I.J., Piva M., Moriceau G., Zangle T.A., Lo R.S., Teitell M.A. (2018). High-Speed Live-Cell Interferometry: A New Method for Quantifying Tumor Drug Resistance and Heterogeneity. Anal. Chem..

[27] Bokemeyer A., Tepasse P.R., Quill L., Lenz P., Rijcken E., Vieth M., Ding N., Ketelhut S., Rieder F., Kemper B. (2019). Quantitative Phase Imaging Using Digital Holographic Microscopy Reliably Assesses Morphology and Reflects Elastic Properties of Fibrotic Intestinal Tissue. Sci. Rep..

[28] Mugnano M., Memmolo P., Miccio L., Merola F., Bianco V., Bramanti A., Gambale A., Russo R., Andolfo I., Iolascon A. (2018). Label-Free Optical Marker for Red-Blood-Cell Phenotyping of Inherited Anemias. Anal. Chem..

[29] Kühn J., Shaffer E., Mena J., Breton B., Parent J., Rappaz B., Chambon M., Emery Y., Magistretti P., Depeursinge C. (2013). Label-free cytotoxicity screening assay by digital holographic microscopy. Assay Drug Dev. Technol..

[30] Makdasi E., Laskar O., Milrot E., Schuster O., Shmaya S., Yitzhaki S. (2019). Whole-Cell Multiparameter Assay for Ricin and Abrin Activity-Based Digital Holographic Microscopy. Toxins.

[31] Mugnano M., Memmolo P., Miccio L., Grilli S., Merola F., Calabuig A., Bramanti A., Mazzon E., Ferraro P. (2018). In vitro cytotoxicity evaluation of cadmium by label-free holographic microscopy. J. Biophotonics.

[32] Mues S., Antunovic J., Ketelhut S., Kemper B., Schnekenburger J. (2016). Novel Optical Approaches for Label.-Free Quantification of Nano-cytotoxic Effects.

[33] Rappaz B., Cano E., Colomb T., Kühn J., Depeursinge C., Simanis V., Magistretti P.J., Marquet P. (2009). Noninvasive characterization of the fission yeast cell cycle by monitoring dry mass with digital holographic microscopy. J. Biomed. Opt..

[34] Popescu G., Park Y., Lue N., Best-Popescu C., Deflores L., Dasari R.R., Feld M.S., Badizadegan K. (2008). Optical imaging of cell mass and growth dynamics. Am. J. Physiol. Cell Physiol..

[35] Kemper B., von Bally G. (2008). Digital holographic microscopy for live cell applications and technical inspection. Appl. Opt..

[36] Gravier J., Garcia F.P.N.Y., Delmas T., Mittler F., Couffin A.-C., Vinet F., Texier-Nogues I. (2011). Lipidots: Competitive organic alternative to quantum dots for in vivo fluorescence imaging. J. Biomed. Opt..

[37] Jacquart A., Keramidas M., Vollaire J., Boisgard R., Pottier G., Rustique E., Mittler F., Navarro F.P., Boutet J., Coll J.L. (2013). LipImage 815: Novel dye-loaded lipid nanoparticles for long-term and sensitive in vivo near-infrared fluorescence imaging. J. Biomed. Opt..

[38] Sayag D., Cabon Q., Texier I., Navarro F.P., Boisgard R., Virieux-Watrelot D., Carozzo C., Ponce F. (2016). Phase-0/phase-I study of dye-loaded lipid nanoparticles for near-infrared fluorescence imaging in healthy dogs. Eur. J. Pharm. Biopharm..

[39] Vauthier C., Dubernet C., Fattal E., Pinto-Alphandary H., Couvreur P. (2003). Poly(alkylcyanoacrylates) as biodegradable materials for biomedical applications. Adv. Drug Deliv. Rev..

[40] Sulheim E., Iversen T.G., To Nakstad V., Klinkenberg G., Sletta H., Schmid R., Hatletveit A.R., Wagbo A.M., Sundan A., Skotland T. (2017). Cytotoxicity of Poly(Alkyl Cyanoacrylate) Nanoparticles. Int. J. Mol. Sci..

[41] Øverbye A., Torgersen M.L., Sønstevold T., Iversen T.G., Mørch Ý., Skotland T., Sandvig K. (2021). Cabazitaxel-loaded poly(alkyl cyanoacrylate) nanoparticles: Toxicity and changes in the proteome of breast, colon and prostate cancer cells. Nanotoxicology.

[42] Kastl L., Isbach M., Dirksen D., Schnekenburger J., Kemper B. (2017). Quantitative phase imaging for cell culture quality control. Cytom. Part. A.

[43] Kemper B., Barroso Á., Eder K., Marzi A., Ritz S., Schnekenburger J., Ketelhut S. (2021). Reduction of the Influence of Parasitic Interferences and Coherence Induced Disturbances in Digital Holography-Based Quantitative Phase Microscopy by Modulation of the Object Illumination with an Electrically Focus Tunable Lens.

[44] Min J., Yao B., Trendafilova V., Ketelhut S., Kastl L., Greve B., Kemper B. (2019). Quantitative phase imaging of cells in a flow cytometry arrangement utilizing Michelson interferometer-based off-axis digital holographic microscopy. J. Biophotonics.

[45] Sternberg S.R. (1983). Biomedical Image Processing. Computer.

[46] Barer R. (1957). Refractometry and Interferometry of Living Cells. J. Opt. Soc. Am..

[47] Jedrzejczak-Silicka M., Mijowska E. (2017). General Cytotoxicity and Its Application in Nanomaterial Analysis. Cytotoxicity.

[48] Barer R. (1953). Determination of dry mass, thickness, solid and water concentration in living cells. Nature.

[49] Barer R., Tkaczyk S. (1954). Refractive Index of Concentrated Protein Solutions. Nature.

[50] Zhao H., Brown P.H., Schuck P. (2011). On the distribution of protein refractive index increments. Biophys. J..

[51] Dunigan D.D., Waters S.B., Owen T.C. (1995). Aqueous soluble tetrazolium/formazan MTS as an indicator of NADH- and NADPH-dependent dehydrogenase activity. Biotechniques.

[52] Eder K., Marzi A., Barroso Á., Kemper B., Schnekenburger J. (2021). Medical Nanoparticle Impact on Macrophage Temporal Dry Mass Development Quantified In Vitro by Digital Holographic Microscopy.

[53] Pavillon N., Kühn J., Moratal C., Jourdain P., Depeursinge C., Magistretti P.J., Marquet P. (2012). Early cell death detection with digital holographic microscopy. PLoS ONE.

[54] Bardyn M., Rappaz B., Jaferzadeh K., Crettaz D., Tissot J.D., Moon I., Turcatti G., Lion N., Prudent M. (2017). Red blood cells ageing markers: A multi-parametric analysis. Blood Transfus..

[55] Mölder A., Sebesta M., Gustafsson M., Gisselson L., Wingren A.G., Alm K. (2008). Non-invasive, label-free cell counting and quantitative analysis of adherent cells using digital holography. J. Microsc..

[56] Rezaei M., Cao J., Friedrich K., Kemper B., Brendel O., Grosser M., Adrian M., Baretton G., Breier G., Schnittler H.J. (2018). The expression of VE-cadherin in breast cancer cells modulates cell dynamics as a function of tumor differentiation and promotes tumor-endothelial cell interactions. Histochem. Cell Biol..

[57] Eder K., Kutscher T., Marzi A., Barroso Á., Schnekenburger J., Kemper B. (2021). Automated Detection of Macrophages in Quantitative Phase Images by Deep Learning Using a Mask Region.-Based Convolutional Neural Network.

[58] Girshovitz P., Shaked N.T. (2012). Generalized cell morphological parameters based on interferometric phase microscopy and their application to cell life cycle characterization. Biomed. Opt. Express.

[59] Belashov A.V., Zhikhoreva A.A., Belyaeva T.N., Salova A.V., Kornilova E.S., Semenova I.V., Vasyutinskii O.S. (2021). Machine Learning Assisted Classification of Cell Lines and Cell States on Quantitative Phase Images. Cells.

