# Supplementary material for: Label-Free Digital Holographic Microscopy for In Vitro Cytotoxic Effect Quantification of Organic Nanoparticles
Source: Cells. 2022 Feb 12;11(4):644. doi: 10.3390/cells11040644 (PMC8870653; doi:10.3390/cells11040644)
Supplement: Supplementary file 1 [file cells-11-00644-s001.zip › cells-1530258-supplementary.pdf]

Article

Label-free Digital Holographic Microscopy for *in vitro* Cytotoxic Effect Quantification of Medical Nanoparticles

Kai Moritz Eder[1,±], Anne Marzi[1,±], Álvaro Barroso[1], Steffi Ketelhut[1], Björn Kemper[1, ‡*] and Jürgen Schnekenburger[1,‡*]

[1] Biomedical Technology Center, University of Muenster, Mendelstraße 17, Muenster, D-48149, Germany

[±], [‡] Contributed equally to this article

* Correspondence: bkemper@uni-muenster.de, schnekenburger@uni-muenster.de

Supplementary materials

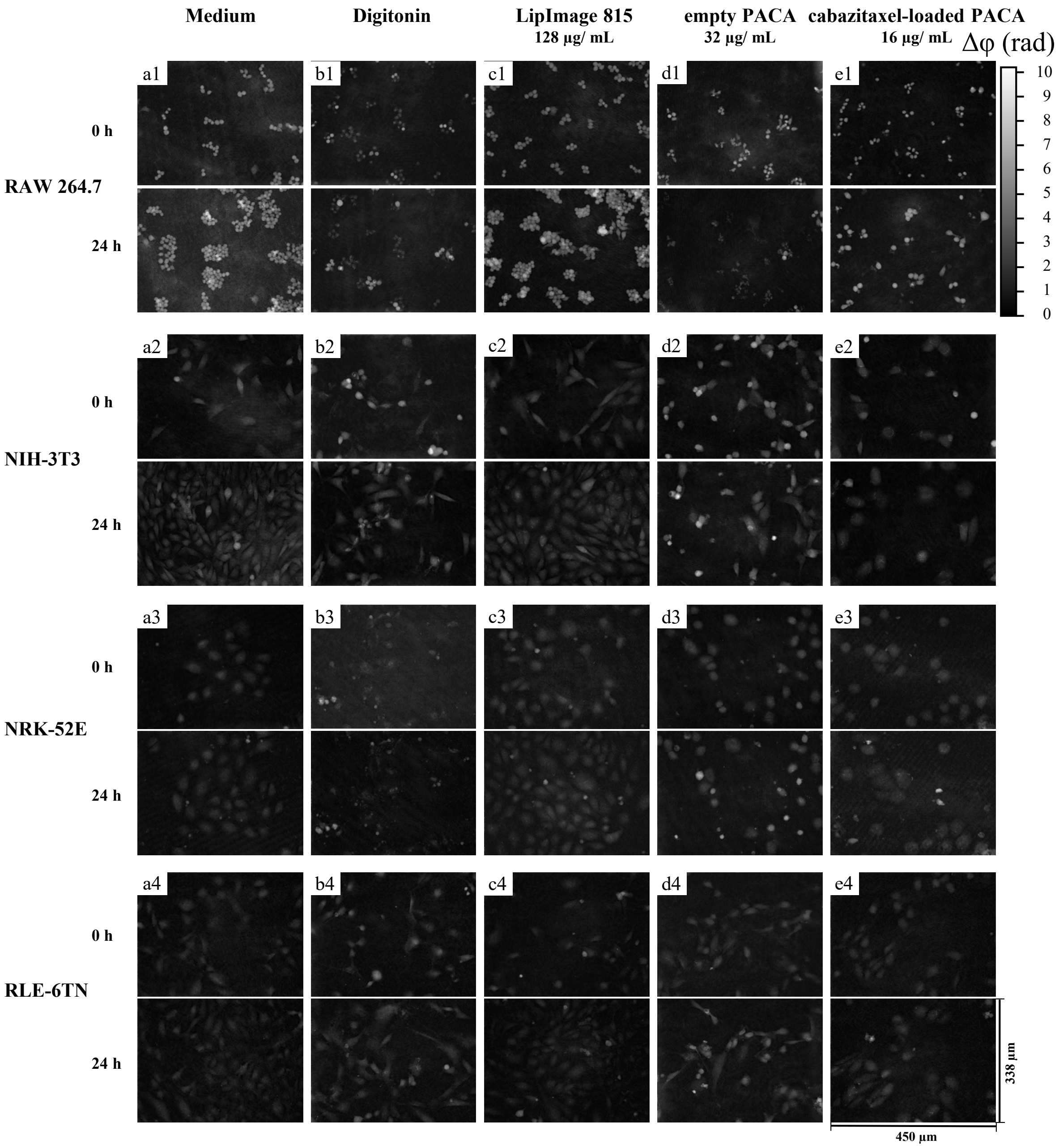

Figure S1. Representative DHM QPI images of the four cell lines incubated with controls and nanoparticles at time points t = 0 h and t = 24 h. To reduce coherence induced image disturbances an electrically tunable lens was used to modulate the object wave while 15 holograms were recorded. Subsequently, from the 15 reconstructed QPI images an average image was calculated. (a1-a4): Normal proliferation documented in DHM QPI experiments of cell culture medium control cells. (b1-b4): Phase images of cytotoxicity control digitonin incubated cells. Cell proliferation was inhibited from time point t = 0 h to t = 24 h. (c1-c4): QPI images of the cell lines incubated with 128 µg/mL of LipImage™ 815 lipidots®. (d1-d4): QPI images of cells incubated with 32 µg/mL of empty PACA nanoparticles. (e1-e4) QPI images of the four cell lines incubated with 16 µg/mL of cabazitaxel-loaded PACA nanoparticles. Gray level representation of quantitative phase images was normalized to the phase range of the entire image data set.

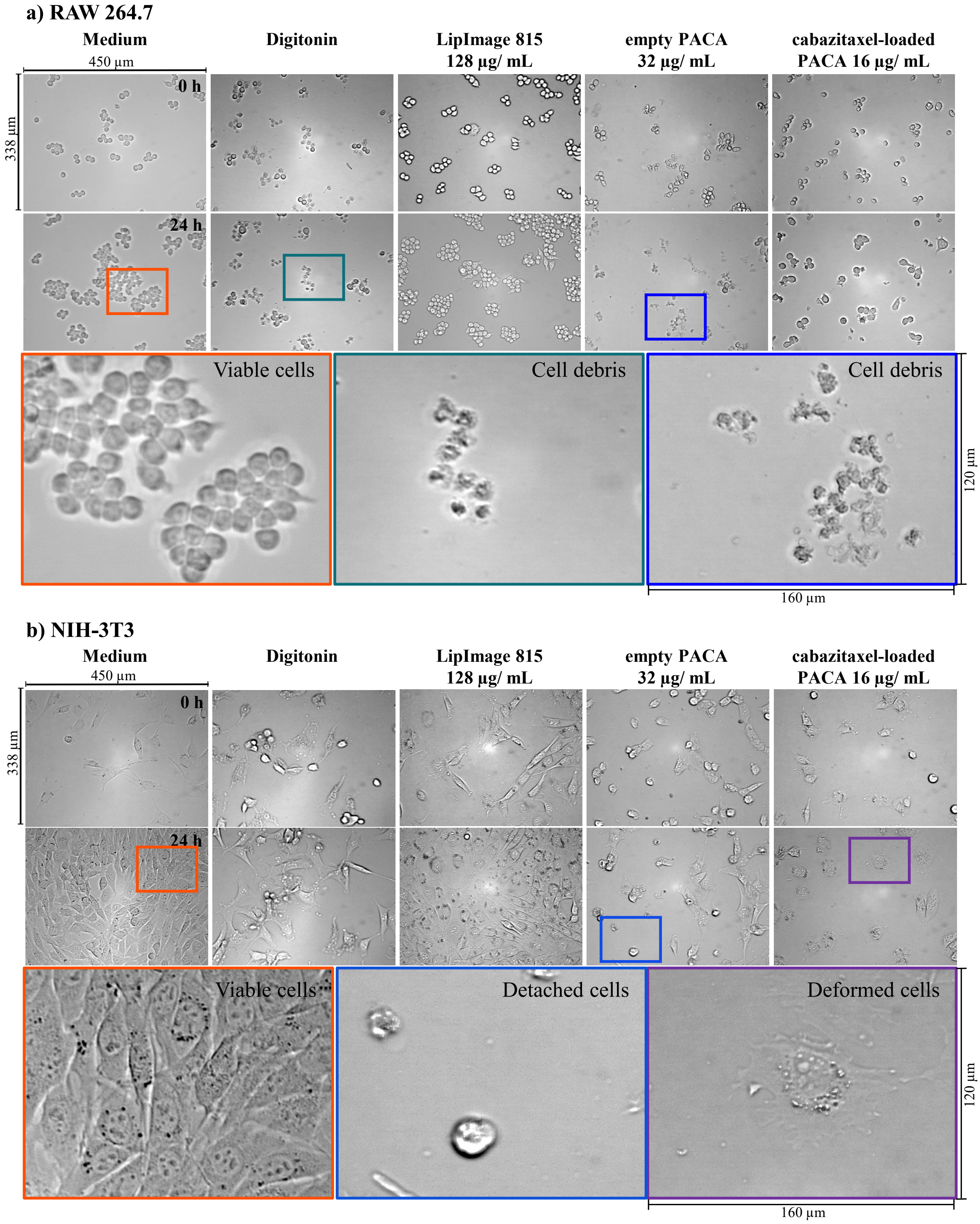

Figure S2. Representative bright-field images of RAW 264.7 macrophages (a) and NIH-3T3 fibroblasts (b) incubated with controls and nanoparticles at time points t = 0 h and t = 24 h. For both cell lines cell culture medium control and cytotoxicity control digitonin is shown. In the digitonin controls cell proliferation was inhibited from time point t = 0 h to t = 24 h. Orange boxes show enlarged areas with viable cells in cell culture medium control. Enlarged areas in green boxes indicate digitonin induced cell debris of lysed RAW 264.7 macrophages. Cells incubated with 128 µg/mL of LipImage™ 815 lipidots® nanoparticles proliferated and grew to a similar extend as observed in the cell culture medium control. For cells incubated with 32 µg/mL of empty PACA nanoparticles cell proliferation was inhibited. Detached cells are shown in the blue framed enlarged image areas. Incubation with 16 µg/mL of cabazitaxel-loaded PACA nanoparticles caused cell deformation, indicated in the enlarged purple framed area, and similar to the PACA nanoparticles, proliferation was inhibited.

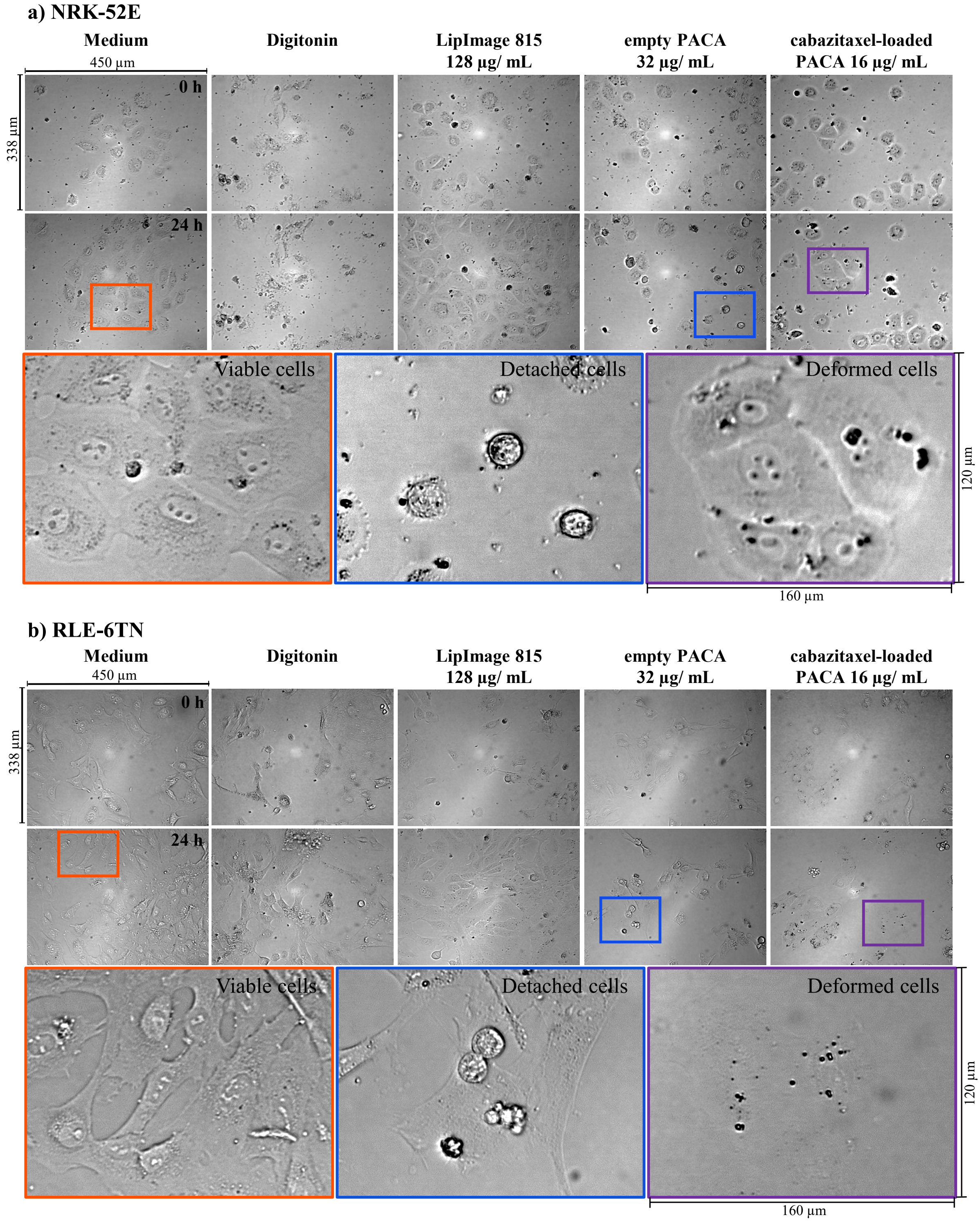

Figure S3. Representative bright-field images of NRK-52E cells (a) and RLE-6TN cells (b) incubated with controls and nanoparticles at time points t = 0 h and t = 24 h. For both cell lines cell culture medium control and cytotoxicity control digitonin is shown. In digitonin controls cell proliferation was inhibited during the observation period 24 h. Enlarged areas framed in orange show viable cells in cell culture medium control. Cells incubated with 128 µg/mL of LipImage™ 815 lipidots® nanoparticles proliferated and grew to a similar extend as observed in the cell culture medium control. Incubation with 32 µg/mL of empty PACA nanoparticles, caused inhibited cell proliferation and detached cells are shown in blue framed enlarged image areas. Incubation with 16 µg/mL of cabazitaxel-loaded PACA nanoparticles caused deformation of cells as indicated in the purple framed enlarged image area and proliferation was inhibited.

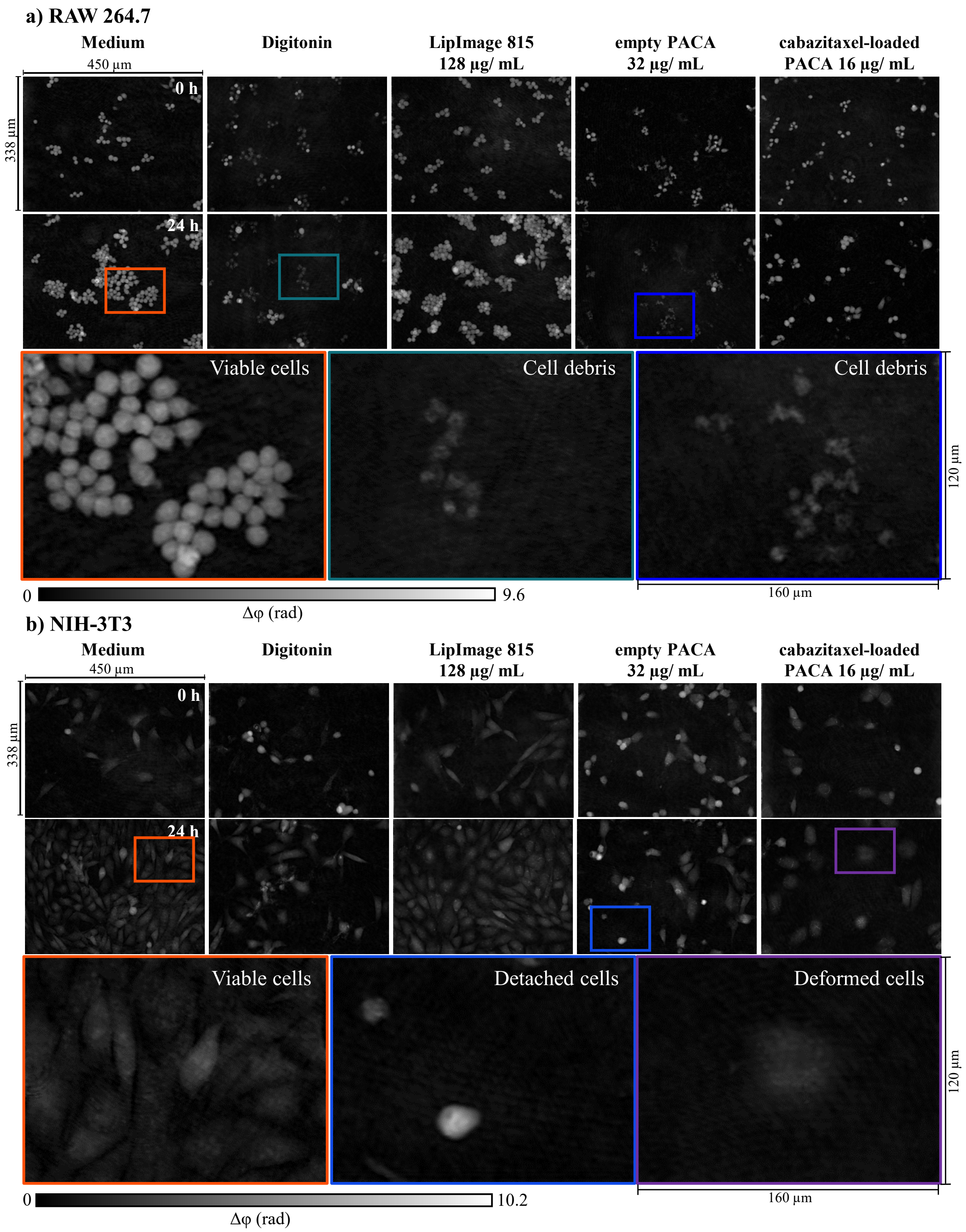

Figure S4. Representative DHM QPI images in gray level representation of RAW 264.7 macrophages (a) and NIH-3T3 fibroblasts (b) incubated with controls and nanoparticles at time points t = 0 h and t = 24 h. For both cell lines cell culture medium control and cytotoxicity control digitonin is shown. In the digitonin controls cell proliferation was inhibited from time point t = 0 h to t = 24 h. Orange boxes show enlarged areas with viable cells in cell culture medium control. Enlarged areas in green boxes indicate digitonin induced cell debris of lysed RAW 264.7 macrophages. Cells incubated with 128 µg/mL of LipImage™ 815 lipidots® nanoparticles proliferated and grew to a similar extend as observed in the cell culture medium control. For ells incubated with 32 µg/mL of empty PACA nanoparticles cell proliferation was inhibited. Detached cells are shown in the blue framed enlarged image areas. Incubation with 16 µg/mL of cabazitaxel-loaded PACA nanoparticles caused cell deformation, indicated in the enlarged purple framed area, and similar to PACA nanoparticles, proliferation was inhibited.

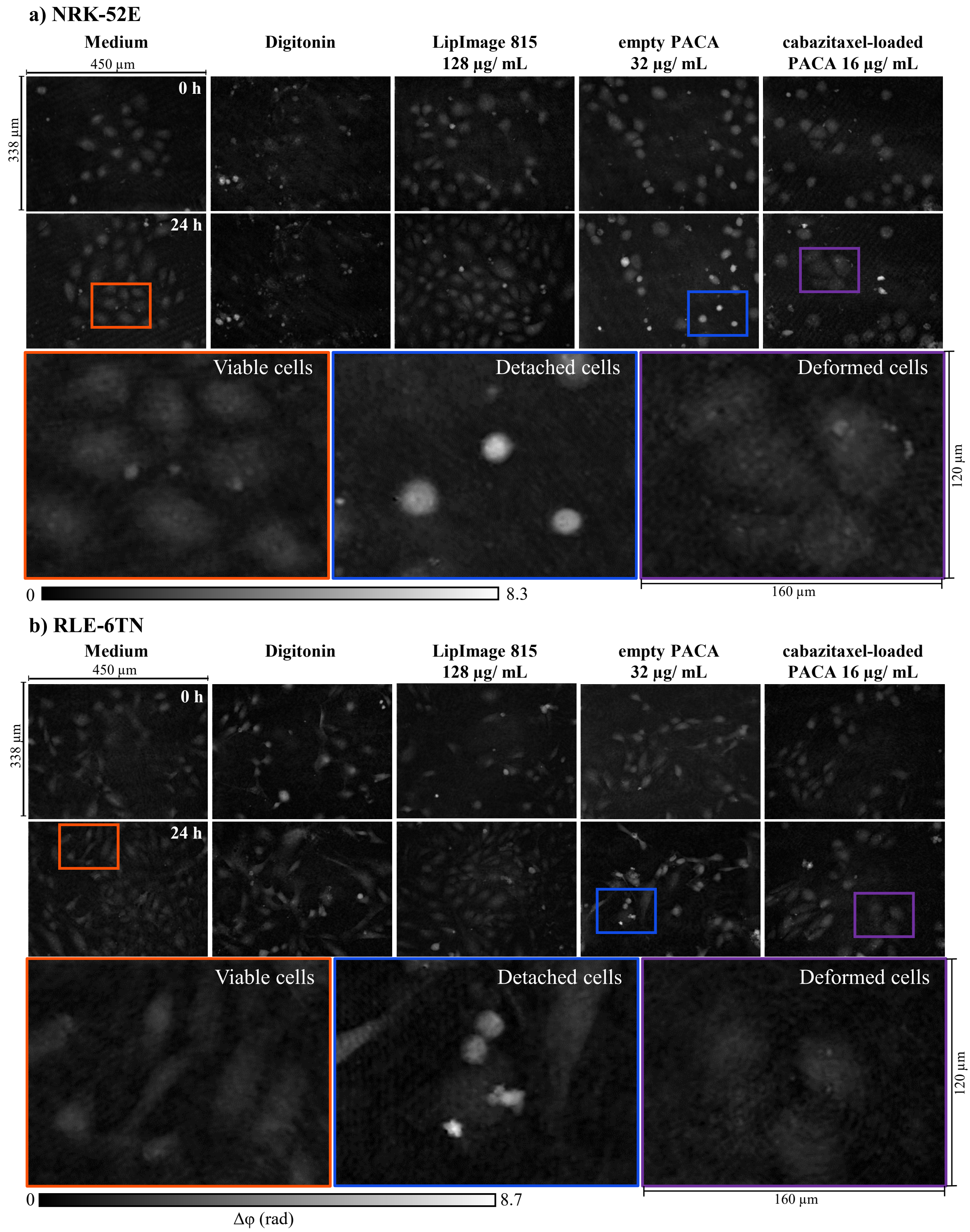

Figure S5. Representative DHM QPI images in gray level representation of NRK-52E cells (a) and RLE-6TN cells (b) incubated with controls and nanoparticles at time points t = 0 h and t = 24 h. For both cell lines cell culture medium control and cytotoxicity control digitonin is shown. In digitonin controls cell proliferation was inhibited during the observation period 24 h. Enlarged areas framed in orange show viable cells in cell culture medium control. Cells incubated with 128 µg/mL of LipImage™ 815 lipidots® nanoparticles proliferated and grew to a similar extend as observed in the cell culture medium control. Incubation with 32 µg/mL of empty PACA nanoparticles, caused inhibited cell proliferation and detached cells are shown in blue framed enlarged image areas. Incubation with 16 µg/mL of cabazitaxel-loaded PACA nanoparticles caused deformation of cells as indicated in the purple framed enlarged image area and proliferation was inhibited.

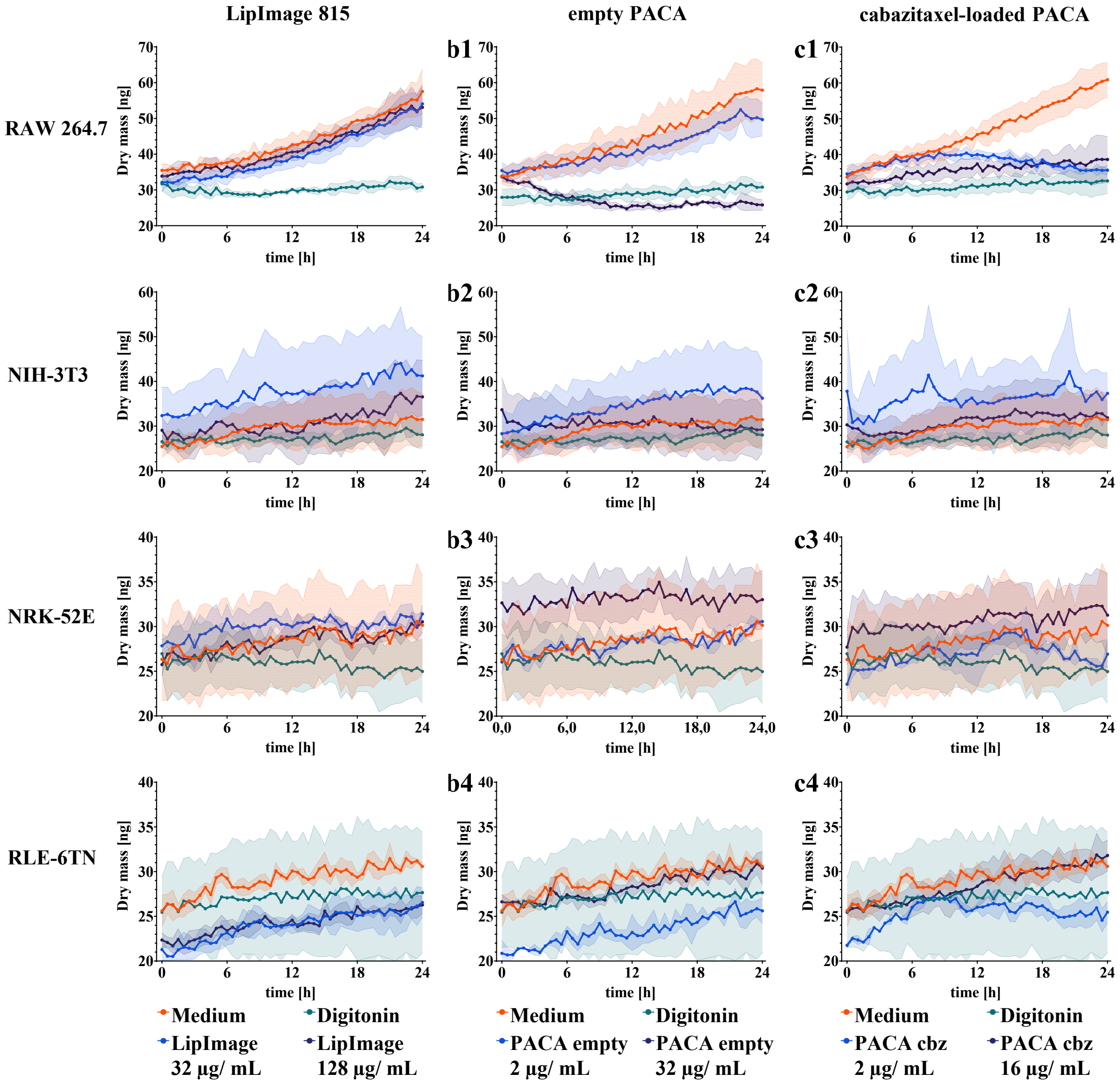

Figure S6. Temporal dry mass development of cell populations with standard deviations indicated in color-coded shadows retrieved from DHM time-lapse measurements for medium control, digitonin cytotoxicity control, and the test set of medical nanoparticles for RAW 264.7, NIH-3T3, NRK-52E, and RLE-6TN cells. Each data point represents the average of dry mass value of n = 12 FOVs that were acquired in N = 3 independent experiments (n = 4 FOVs per independent experiment). Columns (a1-a4): Cell lines after incubation with medium and digitonin cytotoxicity controls, 32 µg/mL and 128 µg/mL of LipImage™ 815 lipidots® nanoparticles. Columns (b1-b4): Cell lines after incubation with controls and 2 µg/mL and 32 µg/mL of empty PACA nanoparticles. Colums (c1-c4): Cell lines after incubation with controls and 2 µg/mL and 16 µg/mL of cabazitaxel-loaded PACA nanoparticles.

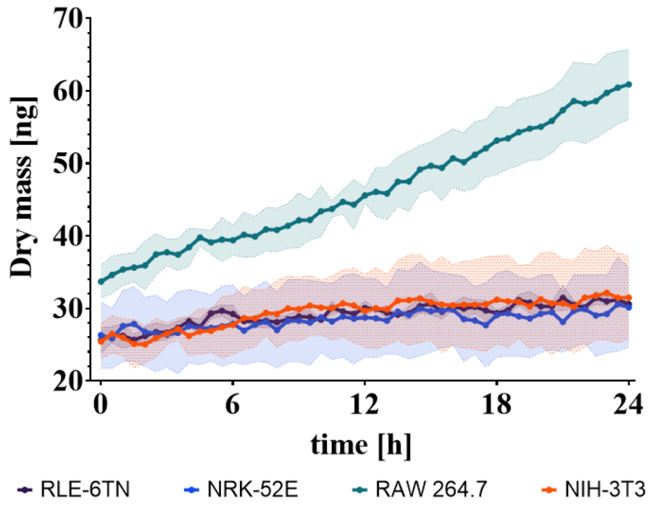

Figure S7. Temporal dry mass development of cell populations with standard deviation indicated in color-coded shadows retrieved from DHM time-lapse measurements for cell culture medium control for RAW 264.7, NIH 3T3, NRK 52E, and RLE 6TN cells. Each data point represents the average of dry mass value of n = 12 FOVs that were acquired in N = 3 independent experiments (n = 4 FOVs per independent experiment). The DHM experiments were performed for 24 h and each FOV was imaged every 30 minutes.
